# Patterns of autoantibody expression in multiple sclerosis identified through development of an autoantigen discovery technology

**DOI:** 10.1172/JCI171948

**Published:** 2025-03-03

**Authors:** Europe B. DiCillo, Evgueni Kountikov, Minghua Zhu, Stefan Lanker, Danielle E. Harlow, Elizabeth R. Piette, Weiguo Zhang, Brooke Hayward, Joshua Heuler, Julie Korich, Jeffrey L. Bennett, David Pisetsky, Thomas Tedder

**Affiliations:** 1 Department of Integrated Immunobiology, Duke University Medical Center, Durham, North Carolina, USA.; 2 EMD Serono, Inc., Boston, Massachusetts, USA.; 3 Departments of Neurology and Ophthalmology, Programs in Neurosciences and Immunology – University of Colorado Anschutz Medical Campus; Aurora, Colorado, USA.; 4 Department of Medicine, Duke University Medical Center and Medical Research Service, Veterans Administration Medical Center, Durham, North Carolina, USA.

**Keywords:** Autoimmunity, Neuroscience, Antigen, Autoimmune diseases, Multiple sclerosis

## Abstract

Multiple sclerosis (MS) is a debilitating autoimmune disease of the CNS, which is characterized by demyelination and axonal injury and frequently preceded by a demyelinating event called clinically isolated syndrome (CIS). Despite the importance of B cells and autoantibodies in MS pathology, their target specificities remain largely unknown. For an agnostic and comprehensive evaluation of autoantibodies in MS, we developed and employed what we believe to be a novel autoantigen discovery technology, the Antigenome Platform. This Platform is a high-throughput assay comprising large-fragment (approximately 100 amino acids) cDNA libraries, phage display, serum antibody screening technology, and robust bioinformatics analysis pipelines. For autoantibody discovery, we assayed serum samples from CIS patients who received either placebo or treatment who were enrolled in the REFLEX clinical trial, which assessed the effects of IFN-β-1a (Rebif) clinical and MRI activity in patients with CIS. Serum autoantibodies from patients with CIS were significantly and reproducibly enriched for known and previously unreported protein targets; 166 targets were selected by over 10% of patients’ sera. Further, 10 autoantibody biomarkers associated with disease activity and 17 associated with patient response to IFN-β-1a therapy. These findings indicate widespread autoantibody production in MS and provide biomarkers for continued study and prediction of disease progression.

## Abstract 2

Antibody specificities in multiple sclerosis are largely unknown; therefore, we used an original and comprehensive autoantibody discovery technology to identify autoantibody specificities in multiple sclerosis

## Introduction

Multiple sclerosis (MS) is a chronic autoimmune disease of the CNS and the most common neurodegenerative disease of people under 40 (1, 2). MS is classified into 3 broad subtypes: (a) relapsing-remitting MS (RRMS), (b) primary progressive MS (PPMS), and (c) secondary progressive MS (SPMS) (3). RRMS typically presents first with an episode of neurologic dysfunction termed clinically isolated syndrome (CIS). Patients with CIS with clinically silent brain MRI lesions or oligoclonal bands are at substantial risk to develop RRMS (4, 5). According to the 2005 McDonald MS criteria, conversion from CIS to definite MS is determined by either a new demyelinating event or MRI evidence of CNS lesions disseminated in time and space (3, 6). As CIS is typically the earliest clinical expression of MS, utilizing serum samples from patients with CIS with known disease outcomes may provide new insights into mechanisms that affect the disease activity in MS. Further, the CIS serum samples collected at Month 0 of the REFLEX clinical trial are from patients who have yet to undergo MS disease–modifying therapies that could affect autoantibody repertoires. Thus, analyzing the autoantibody repertoire of patients with CIS may help identify autoantibodies associated with and possibly contributing to early disease activity and pathological changes in disease.

While studies on MS, especially those in murine models, have posited autoreactive T cells (CD4^+^ and CD8^+^) as the main drivers of pathology (7), the success of anti-CD20 B cell depletion therapies has focused attention on B cells as disease mediators (3). An important role for B cells in MS pathology is supported by studies of the histopathology of lesions in MS and analysis of cerebrospinal fluid (CSF). MS brain tissue demonstrates active inflammatory lesions with B cell infiltrates and complement deposition in some patients (8) and extensive meningeal inflammation associated with cortical pathology in SPMS (3, 9). Immunoglobulins in the CSF (oligoclonal bands) are a diagnostic hallmark of MS and are associated with poorer disease prognosis (10). The beneficial effects of plasmapheresis in 40%–90% of patients who are unresponsive to corticosteroids provide additional indirect evidence for the role of autoantibodies in MS (11). The specificity of MS antibodies and oligoclonal bands, however, remain poorly defined. Although some autoantigen targets (e.g., KIR4.1, MBP, and a glial protein cross reactive with EBNA1) have been proposed, validation is lacking (12) with few exceptions (13).

The FDA has approved over 20 disease-modifying therapies for treating MS that vary in their mechanism of action; 6 are approved for CIS. Subcutaneous IFN-β-1a (Rebif) gained regulatory approval from the FDA for CIS following the Phase 3 REFLEX (Rebif FLEXible dosing in early MS) trial demonstrating its efficacy (14–16). This placebo-controlled trial enrolled 517 patients with CIS at high risk for McDonald MS conversion and determined responses with MRI imaging. Conducted over a period of 2 years, this study showed that Rebif lowers conversion risk by approximately 50%. Studies have not yet established the mechanism of action for IFN-β-1a treatment, although in vitro studies with B cells from patients with MS indicate the modulation of B cell activity. Specifically, these studies show that treatment reduces MHC antigen presentation by B cells, lowers inflammatory cytokine production, and increases antiinflammatory cytokine production (10, 17, 18).

Given the functions of B cells and their products and the activity of IFN on B cells, we hypothesize that autoantibodies may contribute to the pathogenesis of MS and could provide biomarkers for disease activity and treatment responses. We further hypothesize that comprehensively defining autoantibody targets in CIS/MS may elucidate the autoimmune landscape promoting pathogenesis. Moreover, we hypothesize that autoantibody profiles, e.g., combinations of specificities, rather than a single autoantibody target may be required for defining serological biomarkers, considering prior difficulties in the field. Irrespective of a direct link of antibodies to disease manifestations, autoantibody profiles may serve to predict disease course and monitor treatment response in a precision medicine approach.

To investigate these possibilities, we assayed MS sera with an original technology, termed the Antigenome Platform, which we developed for in-depth discovery of autoantigens. The Antigenome Platform is a cutting-edge high-throughput assay that pairs human cell line–derived cDNA fragments, phage display (19), a serum antibody selection system, and a bioinformatics pipeline to survey diverse autoantibody reactivities. Since the probability of success in cloning large in-frame cDNA fragments is generally low (e.g., 1 in 1.3 × 10^6^ for 100 amino acids), we strategically developed methods to overcome this limitation. The Platform utilizes bead-based size selection to exclude short transcripts. In addition, a 2-plasmid sequential cloning system excludes stop-codon–containing fragments by first requiring cDNA fragment read through to confer antibiotic resistance and then requiring read through for phage production.

The use of the Antigenome Platform extends prior studies on autoantigen discovery by assessing serum IgG antibody binding to large protein fragments (up to 250 amino acids) of approximately 90% of human genes. Prior efforts to uncover autoantibodies in MS have predominantly focused on low molecular weight peptides or assessed brain/myelin antigens exclusively (20). In contrast, our methods assess potential autoantigens from a broader set of tissues or cells. The study herein describes the development of the Antigenome Platform and its application in a retrospective subgroup analysis of REFLEX trial serum samples (14–16). We present data on the performance characteristics of the Antigenome Platform and the derivation of autoantibody profiles associated with CIS, MS disease activity, and response to IFN-β-1a. Together, these findings suggest that autoantibodies can be used as MS biomarkers in conjunction with clinical and imaging assessment, and may help elucidate the pathogenesis of MS.

## Results

### A stringent 2-plasmid selection system for large in-frame cDNA library construction.

**Figure 1:**
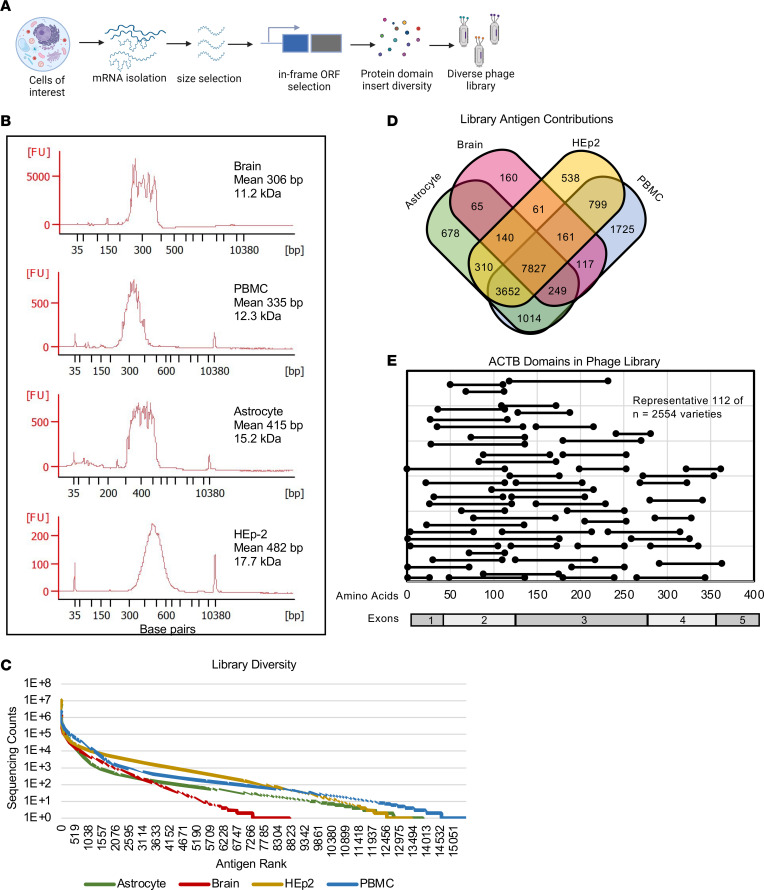
Genome-wide protein fragment expression library generation. (**A**) Expression library construction. mRNA purified from HEp-2 cells, astrocytes, brain white matter, and PBMCs was fragmented to generate ORF transcripts encoding domain-sized protein fragments. > 1 × 10^6^ distinct in-frame transcripts were cloned for phage library construction. Individual libraries were proportionally pooled. (**B**) Library cDNA insert size distributions. Histograms show cDNA insert sizes (base pairs) of the 4 final expression libraries. (**C**) Sequencing count values (log_10_ scale, *y*-axis) represent the relative number of annotated genes in each library. The *x*-axis shows ranking of each gene from highest counts to lowest counts relative to each library. (**D**) Venn diagram showing the number of unique proteins each cell type contributes to the total transcript library. (**E**) Representative protein fragments of the ACTB gene expressed within the pooled cDNA library after selection. Black lines represent expressed library protein fragments of the ACTB protein. The 112 fragments shown represent 2,554 total ACTB fragments in the library.

The Antigenome Platform is a plasmid-based selection system developed to identify targets of autoantibody reactivity across the human cDNA–derived proteome. This technology uses libraries of in-frame, high-diversity, protein domain–sized fragments generated from human genome–encoded transcripts to obtain a broad coverage of target autoantigens agnostically. The transcript sources include HEp-2 cells, brain white matter, cultured astrocytes, and PBMCs (Figure 1A), which were chosen to maximize the discovery of CNS autoantigens and antinuclear antigens. HEp-2 cells are a human epithelial cell line used for assaying antinuclear antibodies, for which some patients with MS are positive (21, 22).

We generated libraries using a stringent 2-plasmid selection system. The first plasmid requires an in-frame open reading frame (ORF) to confer antibiotic resistance. After antibiotic selection, the resulting in-frame ORFs are cloned into the second plasmid (phagemid). The phagemid requires an in-frame ORF for M13 phage protein production. As such, this approach markedly reduces phagemids containing out-of-frame, non-ORF inserts. Other systems for autoantigen discovery in MS have led to the identification of out-of-frame proteins as targets (23). We validated this approach by Sanger sequencing 266 randomly isolated phagemids, which revealed 74% ORFs and 77% in-frame and absent of stop codons (Supplemental Table 1; supplemental material available online with this article; https://doi.org/10.1172/JCI171948DS1). Over 99% of inserts were in the correct orientation (data not shown). The average cDNA size ranged from 306 bp (brain library) to 482 bp (HEp-2 library) (Figure 1B). The large fragment size (mean of approximately 14.1 kDa) suggests the representation of conformational and linear epitopes. The display of conformations is important because an estimated 90% of protein epitopes recognized by antibody molecules are conformational (24); furthermore, pathogenic autoantibodies recently identified in MS CSF were found to be conformation dependent (13).

The 4 cell-derived libraries were equally pooled by phage number to create the final library. Wherein, 46% of phage inserts encoded known proteins; the majority remaining were ribosomal RNA sequences. Each phage library was sequenced with next-generation sequencing to evaluate protein diversity (Figure 1C). The astrocyte, HEp-2, PBMC, and brain white matter libraries represent at least 13,219, 12,406, 14,925, and 9,561 human proteins, respectively. In total, expression library complexity includes approximately 18,000 human proteins, with each cell type contributing between 139–1,784 unique proteins (Figure 1D).

Data analysis indicates that an average of 80 unique protein fragments represent each protein (range of 1–38,698), totaling more than 1 × 10^5^ unique protein fragments. This representation is demonstrated by results with the β-actin protein (ACTB) (Figure 1E).

### Reproducibility and specificity of the Antigenome Platform.

**Figure 2:**
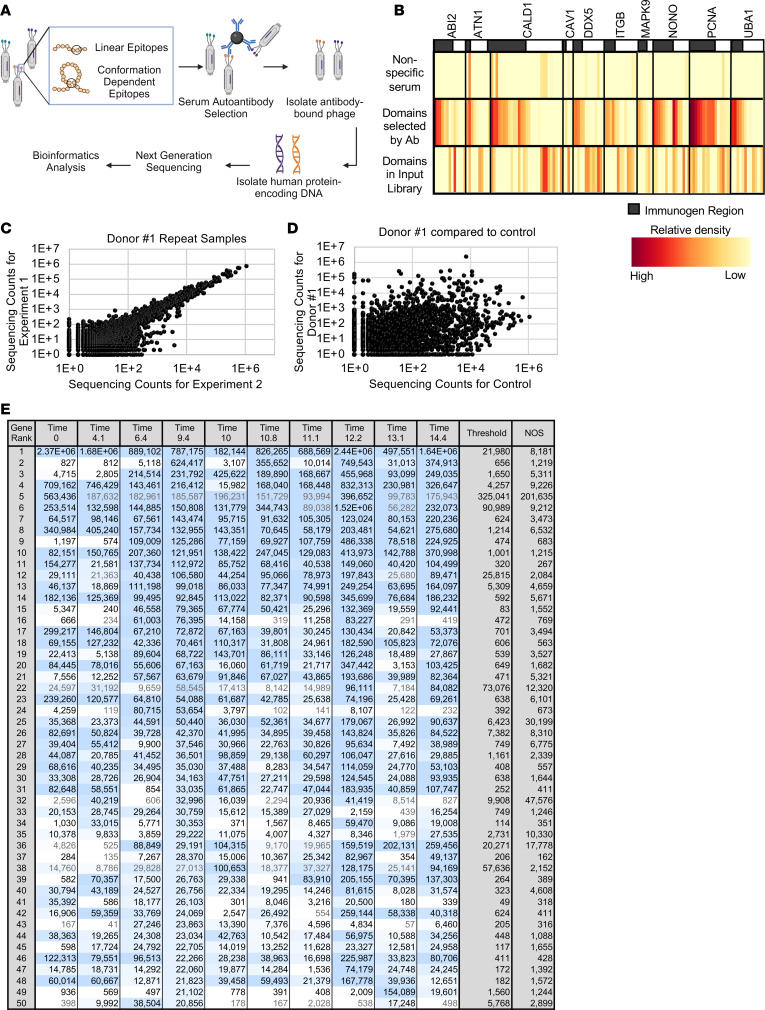
Autoantigen selection assay. (**A**) Schema for autoantigen selection. Phage displaying human protein fragments were immunoselected by serum IgG-bound protein G-paramagnetic beads. Samples were multiplexed, identified by deep sequencing, quantified, and statistically analyzed. (**B**) Immunoselection results for 9 rabbit antisera generated against specific protein fragments of the human proteins ABI2, ATN1, CALD1, DDX5, ITGB1, MAPK9, NONO, PCNA, and UBA1. Black boxes indicate immunogen region (target protein fragments); white boxes indicate nonimmunogen region (off-target protein fragments) of the same protein. Protein fragments selected by the rabbit antisera antibody (Ab), fragments selected by a control serum, and the relative density of fragments available for selection in the input library are all shown. (**C** and **D**) Scatter plot showing autoantigens (dots) selected in 2 independent experiments using (**C**) the same serum sample (Donor #1) or (**D**) 2 different serum samples (Donor #1 and an age-matched control). Axes indicate sequencing counts (log_10_ scale). (**E**) Heatmap shows autoantigens selected by Donor #1 sera over the span of 14.4 years, where time 0 is the first serum sample collected. Each row represents a different antigen, ranked based on time 9.4 serum autoantibody selections. Only the top 50 autoantigens (based on counts) selected by Donor #1 time 9.4 are shown for brevity.

Human serum samples were incubated with the phage library (1 × 10^10^ phage; an average of 574,218 phage per protein) and protein G–conjugated paramagnetic beads to isolate serum IgG-bound phage clones. DNA encoding selected protein fragments was isolated and identified by next-generation sequencing. Sequencing counts were mapped to the human genome with a bioinformatics pipeline powered by the STAR alignment tool (25) (Figure 2A). Input library and a human serum sample repeat (Donor #1) were included in every experiment to detect and correct any batch effects and maintain high reproducibility standards.

We used antibodies with previously defined protein targets to assess selection specificity. For these studies, rabbit antisera generated against human protein fragments were purchased and assayed with the Antigenome Platform. The appropriate immunogen fragments were specifically selected by each rabbit antisera assayed, even though nonimmunogen fragments of the same protein had higher representation in the input library. In contrast, a control serum did not select these fragments (Figure 2B). Thus, selection is antibody specific and appropriately mapped through the bioinformatics pipeline.

The collection of autoantigens selected by sera is sample-specific and highly reproducible among experiments, as demonstrated by the Donor #1 serum sample and an unrelated healthy control (HC) sample. The Donor #1 sample was assayed in 2 independent experiments and achieved high agreement in autoantigen selection, visualized by scatter plot with an R^2^ value of 0.9246 (Figure 2C). On the contrary, an unrelated HC serum sample did not select the same antigens (Figure 2D). These findings suggest specific autoantigen selection rather than assay variability or background noise. The data also suggest that autoantibody profiles may be largely stable over time since there is 96.6% agreement in autoantigen selection among Donor #1 serum samples drawn over 14 years, as measured using binary-transformed data (Figure 2E).

### Sera from patients with CIS assayed with the Antigenome Platform.

Having established that the Platform maps antigen binding, we used serum samples from the REFLEX clinical trial to determine autoantibody profiles for patients with CIS who did not meet the 2005 McDonald MS criteria (6) at the time of trial enrollment. We further assessed the association of these autoantibody profiles with disease activity in placebo or Rebif IFN-β-1a-treated patient subgroups. Patients who converted to McDonald MS during the REFLEX trial were considered to have disease activity due to either (a) new MRI activity fulfilling space and/or time criteria or (b) a new clinical attack (6).

Patients in 4 subgroups were selected: (a) placebo-treated patients without active disease (PBO-NA, *n* = 21), (b) placebo-treated patients with active disease (PBO-A, *n* = 27), (c) Rebif IFN β-1a-treated, non-active (RNF-NA, *n* = 27), and (d) Rebif IFN β-1a-treated, active (RNF-A, n=27). Only a single participant in the “no activity” group was observed to have a new MRI lesion considered no-diagnostic for MS. To select patients from the 4 subgroups with balanced baseline demographic and disease characteristics, propensity score (PS) matching was applied. Distributions of potential confounders after PS matching for patients in the different subgroups indicate adequate matching (Supplemental Figure 1). Sera collected from 3 time points (month 0 [start of trial], month 6, and month 24) were assayed, totaling 306 serum samples from 102 patients. A total of 43 HC serum samples were assayed for comparison. The Input Library sequencing control was highly reproduced among batches, suggesting minimal batch effect (Supplemental Figure 2).

### Autoantibody targets associated with CIS compared with HC samples.

**Figure 3:**
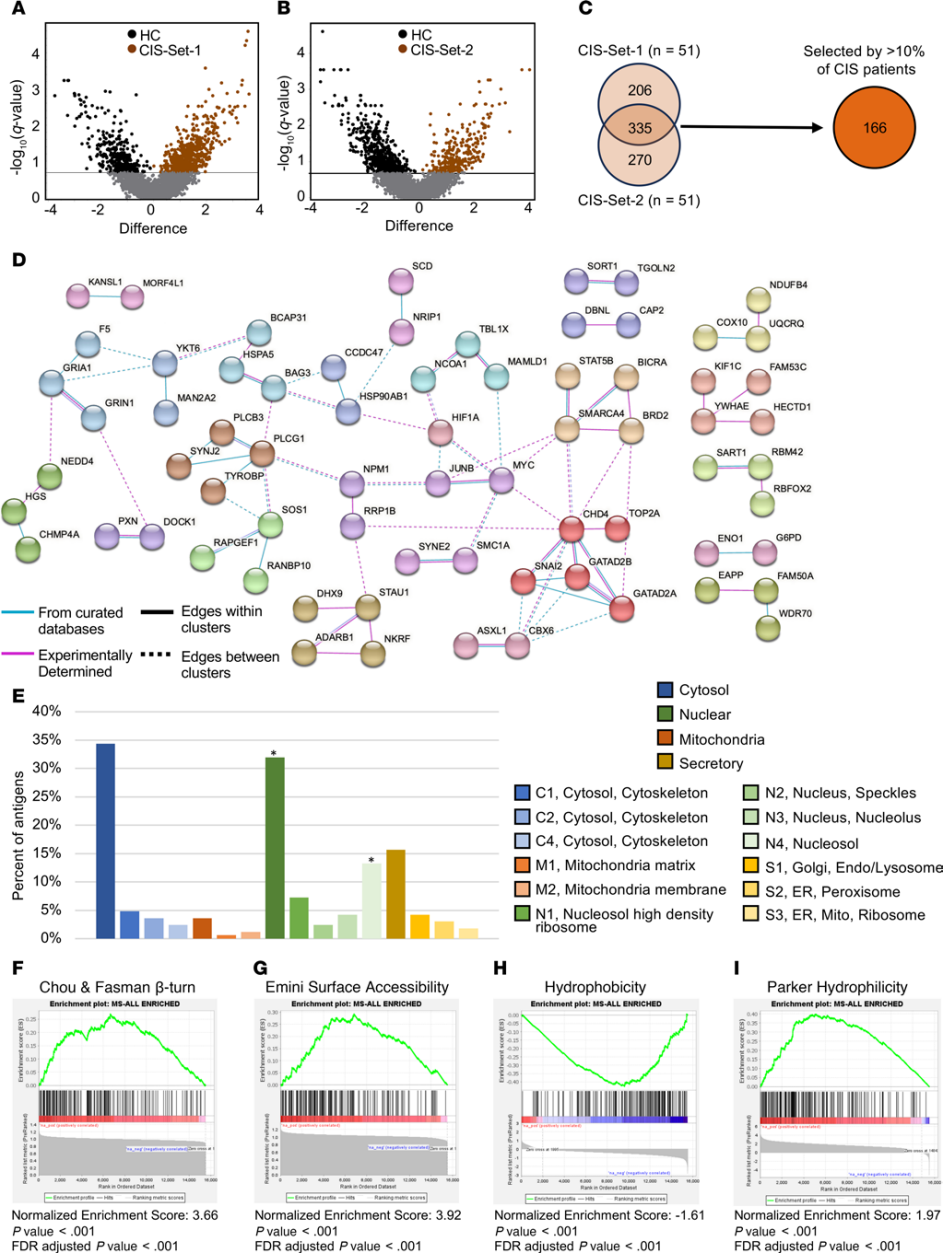
Autoantigen specificities selected by sera from patients with CIS. (**A** and **B**) Individual autoantigens (dots) are statistically compared between the HC group (*n* = 43) and (**A**) The test group of patients with CIS (CIS-Set-1; *n* = 51; month 0), (**B**) The validation group of patients with CIS (CIS-Set-2; *n* = 51; month 0). The *x*-axis represents the difference in sequencing count means between cohorts taken from log_2_-transformed sequencing counts. The *y*-axis represents the –log_10_ of *q* values (higher values indicate greater significance). Autoantigens above the horizontal line (*q* = 0.2) are considered significant. (**C**) Venn diagram shows the overlap of autoantigens selected by CIS-Set-1 and CIS-Set-2. The autoantigens reproducibly selected were extracted and filtered for autoantigens selected by at least 10% of all patients with CIS; 166 autoantigens remained. Kruskal-Wallis rank test was used for all statistical comparisons. (**D**) A protein-protein interaction network of the 166 reproducibly selected CIS-associated autoantigens selected by at least 10% of patients. Only interactions derived from STRING “experimental” and “database” data sources are shown. Proteins without shared connections are omitted. Circles represent antigens and the connecting lines represent interactions between antigens. MCL clustering was applied to help distinguish stronger interactions, where each cluster is uniquely colored. Solid lines between connections indicate stronger interactions than dotted lines. (**E**) Subcellular locations of the 166 CIS-associated autoantigens. Charts detail the percent of antigens that are predominately located in each subcellular compartment and subcompartment. Asterisks indicate locations that are significantly enriched (*P* value < 0.05) over what would be expected from a random set of proteins of the same size. Derived from SubCell Barcode (www.subcellbarcode.org). (**F**–**I**) Protein structure properties as labeled. Values are sorted in descending order; the gray curve indicates the values for all proteins included, and the black vertical lines indicate the placement of the 166 CIS-enriched autoantigens in the ranked list. The green curve indicates the enrichment score. The red and blue color gradient represents positive (red) to negative (blue) values.

The assayed patients with CIS (*n* = 102) were divided evenly into test and validation sets (CIS-Set-1 and CIS-Set-2). The 4 subgroups (PBO-A, PBO-NA, RNF-A, and RNF-NA) were evenly distributed between the 2 sets; however, the patients were otherwise randomly distributed. The sequencing counts of autoantigens selected by CIS-Set-1 (*n* = 51) or CIS-Set-2 (*n* = 51) sera at month 0 were each statistically compared with those selected by HC (*n* = 43). To reduce computation time, the approximately 18,000 measured autoantigen targets were filtered to include only those selected by at least 1% of patients (based on binary-transformed data). Filtering resulted in 4,357 and 4,417 statistically compared autoantigen targets for CIS-Set-1 and CIS-Set-2, respectively. The statistical comparisons revealed 863 differentially selected autoantigens between CIS-Set-1 and HCs (541 associating with CIS-Set-1 and 322 associating with HC) and 876 between CIS-Set-2 and HCs (605 associating with CIS-Set-2 and 271 associating with HCs) (Figure 3, A and B, and Supplemental Data File 1). Of the 541 and 605 autoantigens associating with CIS-Set-1 and CIS-Set-2, respectively, 335 autoantigens were reproducibly selected by both sets, 206 autoantigens were selected only by CIS-Set-1, and 270 autoantigens were selected only by CIS-Set-2 (Figure 3C). Only the 335 autoantigens reproducibly selected by patients with CIS were considered to be CIS-associated autoantigens for the continuation of the study. Statistical tests used Kruskal-Wallis Rank Sums Test at a *P* value of less-than 0.05 and FDR-adjusted *P* value (*q* value) of less-than 0.2.

To verify that the selected autoantigens were not biased by their representation in the input library, we ranked all approximately 18,000 autoantigens and evaluated the ranks of the 335 selected autoantigens. These had a median rank of 3,489, ranging from 50–11,993, indicating no selection bias. The selection counts for these 335 CIS-associated autoantigens were enriched over the input library (Supplemental Data File 2). They were positively selected by 1%–79% of patients with CIS (Supplemental Data File 3). For simplicity and to avoid focusing on autoantigens that may be rare, we focused on the 166 autoantigens represented in at least 10% of patients with MS (Figure 3C).

### Protein-protein networks for CIS-associated autoantibody targets.

To determine whether CIS-associated autoantigens target common pathways or complexes, we explored known protein-protein interactions using the STRING database. STRING integrates associations from multiple sources, including experimental databases and computationally predicted interactions (26).

Of the 166 CIS-associated antigens selected by at least 10% of patients with CIS, 72 (43%) interact with other CIS-associated autoantigens, whether physically or functionally (Figure 3D), based on experimental and database-derived data. Physical interactions are predicted to occur among 60 CIS-associated antigens (Supplemental Figure 4A). PLCG1 is the most well-connected hub antigen with connections to 9 other autoantigens (Figure 3D). Three interacting antigens, CHD4, GATAD2B, and GATAD2A are members of the nucleosome remodeling and histone deacetylation (NuRD) complex (also called the Mi-2 complex), suggesting that the NuRD complex may be an autoimmune target in CIS/MS.

### Subcellular locations of CIS-associated autoantibody targets.

To explore further the nature of the CIS-associated autoantigen targets, we used the SubCellBarcode database to identify their subcellular locations. This database uses mass spectrometry and cell fractionation protocols to catalog the dominant subcellular locations of 12,418 proteins (27).

General subcellular location data are available for 142 of 166 CIS-associated autoantigens, and specific subcellular compartment data are available for 81 of 166 (Figure 3E and Supplemental Data File 4). CIS-associated autoantigens are enriched in the nucleus (53 of 166 autoantigens) and N4 (nucleosol) nuclear subcompartment (22 of 166). Moreover, analysis of protein-protein interactions and subcellular locations collectively indicate that some, but not all, autoantibody targets occupy similar spaces or somehow interact.

### Biochemical properties of CIS-associated autoantibody targets.

We next explored whether common biochemical or structural features may play a role in autoantibody targeting. Using the Immune Epitope Database (IEDB) (28) and gene set enrichment analysis (GSEA) (29, 30), we found that the 166 CIS-associated autoantibody targets are significantly enriched for high Chou and Fasman β-turn scores (normalized enrichment score [NES]: 3.66), high Emini surface accessibility (NES: 3.92), high Parker Hydrophilicity (NES: 1.97), low hydrophobicity (NES: –1.61), and a high fraction of amino acids in β-turns (NES: 3.00), each with a *P* value of less-than 0.001 compared with all of the proteins available in the input library. There was no enrichment when comparing isoelectric point or the fraction of amino acids in β sheets (Figure 3, F–I, and Supplemental Figure 3, A–C).

### Tissue expression of CIS-associated autoantibody targets.

Since MS is a CNS disease, autoantigens expressed predominantly in the brain are of particular interest. Most autoantigen targets (115 of 166) found in this study are widely expressed in human tissues, according to Human Protein Atlas (31). However, some autoantigens show tissue enrichment (Tables 1 and 2). Ten autoantigens (6%) are enriched or enhanced in the brain: GRIA1, GRIN1, SORT1, SYNJ2, KCTD17, CBX6, TRAK2, EXPH5, SCD, and KIF1C. Previous studies have also identified GRIN1 as a potential autoantibody target in MS (32–37). Of note, 16 autoantigens (9.6%) have enhanced expression in the skeletal muscle: MYLK3, KIF1C, SHISA4, CD99L2, NEDD4, CAP2, SVIL, COX10, FLII, SSH2, RAPGEF1, SYNE2, BAG3, NDUFB4, HECTD1, and NT5C3A. This result could indicate cell damage and a local immune response occurring in both the brain and muscle.

### Functional annotations of CIS-associated autoantibody targets.

To further investigate CIS-associated autoantigen targets, we used the STRING database to analyze pathway and functional enrichments, including Gene Ontology terms (GO-terms) (Tables 3 and 4 and Supplemental Data File 5). GO-terms groups genes based on shared biological pathways, cellular components, or molecular functions (38). Significant enrichments were found for the “histone deacetylase complex” (including CHD4, GATAD2B, and GATAD2A), “SWI/SNF superfamily type complex,” “nuclear speck,” “actin cytoskeleton,” “cell junction,” brain development processes, and “nucleic acid binding.” These enrichments are crucial for understanding disease processes, especially since cell junctions maintain the blood-brain barrier, which is disrupted in MS. Autoimmunity targeting nucleic acid and chromatin binding proteins is common in many autoimmune diseases (39).

Additionally, there is an enrichment of proteins in the brain, skeletal system, connective tissue, and cancer cells (e.g., leukemia and bone marrow cancer). This suggests a broad immune dysregulation in patients with CIS/MS, not limited to brain proteins, and may indicate an antitumor response controlling metastasis.

### Autoantibody-target profiles associated with disease activity and Rebif treatment.

We investigated whether specific autoantibody profiles were linked to disease activity during the 2-year REFLEX trial in placebo or Rebif-treated subgroups. We statistically compared the 335 CIS-associated autoantigens (Figure 3C) between each MS subgroup (PBO-A, PBO-NA, RNF-A, RNF-NA) and HCs at baseline. Autoantigens not selected by any patients were excluded.

**Figure 4:**
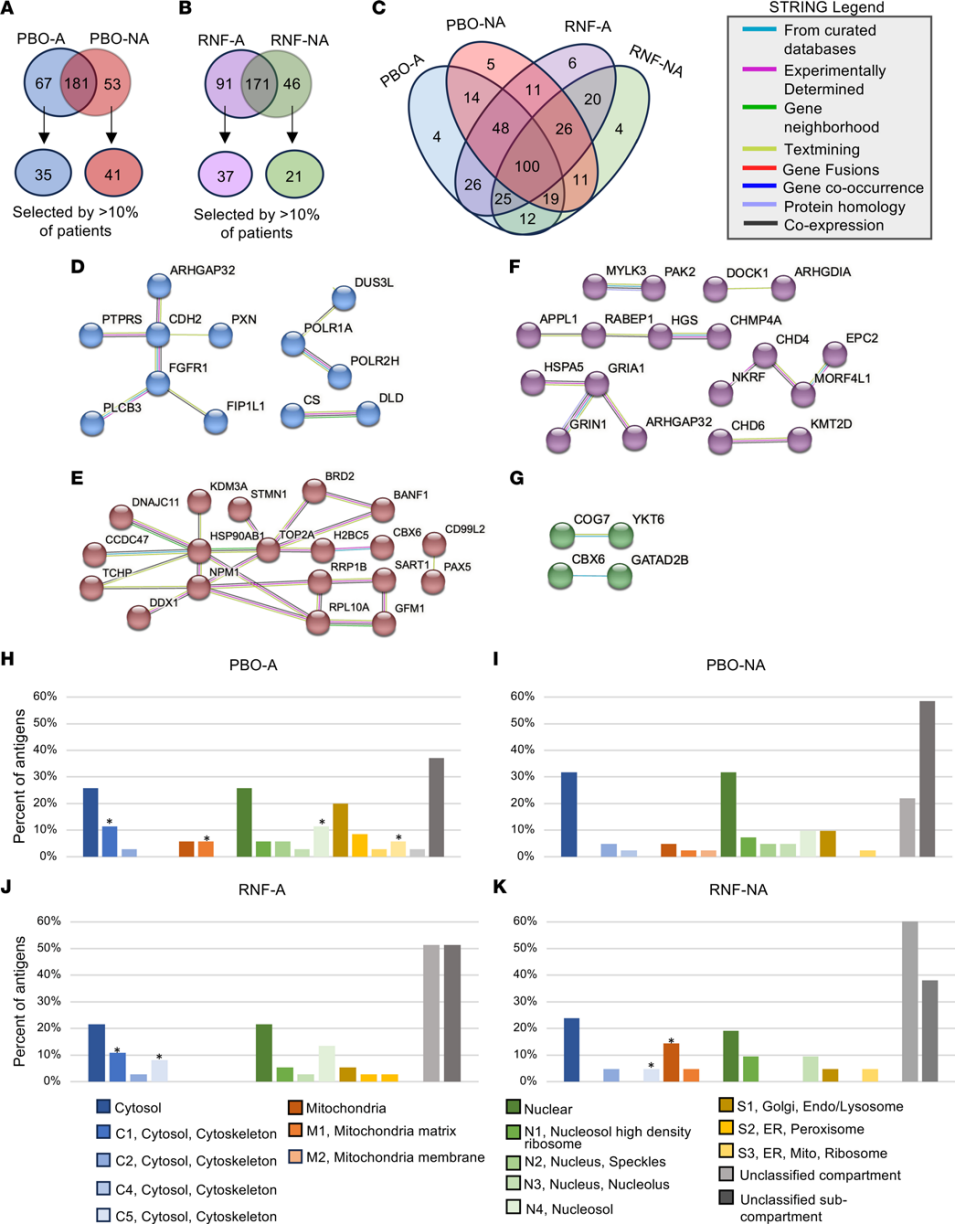
CIS-subgroup–enriched autoantigens. (**A**) Venn diagram shows the overlap of PBO-A– and PBO-NA–enriched autoantigens compared with HCs. The second tier indicates the number of autoantigens selected by more-than 10% of PBO-A (blue) or PBO-NA (red) from the uniquely enriched PBO-A (67) or PBO-NA (53) autoantigens. (**B**) Venn diagram shows the overlap of antigens enriched in the RNF-A and RNF-NA subgroups compared with HCs. The second tier indicates the number of autoantigens selected by more-than 10% of RNF-A (purple) or RNF-NA (green) from uniquely enriched RNF-A (91) or RNF-NA (46) autoantigens. (**C**) Venn diagram shows the overlap of antigens enriched in each MS subgroup compared with HCs. (**D**–**G**) A protein-protein interaction network for (**D**) The 35 PBO-A autoantigens uniquely enriched compared with PBO-NA and shared by more-than 10% of patients, (**E**) The 41 PBO-NA autoantigens uniquely enriched compared with PBO-A, found in more-than 10% of patients, (**F**) The 37 RNF-A autoantigens uniquely enriched compared with RNF-NA, found in more-than 10% of patients, and (**G**) The 21 RNF-NA autoantigens uniquely enriched compared with RNF-A, found in more-than 10% of patients. Circles represent antigens and the connecting lines represent interactions between antigens, colored according to the type of data from which the information is derived. For **D**–**G**, the “STRING Legend” indicates what the color of the connecting lines represent. Disconnected nodes are omitted. Derived from STRING database (string-db.org). (**H**–**K**) Graphs show the percent of antigens in each subcellular location for (**H**) The group of 35 PBO-A–associated autoantigens, (**I**) The group of 41 PBO-NA–associated autoantigens, (**J**) The group of 37 RNF-A–associated autoantigens, and (**K**) The group of 21 RNF-NA–associated autoantigens. Stars denote significantly enriched locations (*P* value < 0.05) over what is expected from the same number of random set of proteins the same size. Derived from SubCell Barcode (www.subcellbarcode.org).

Of the 335 autoantigens, 248 and 234 were significantly associated with PBO-A or PBO-NA compared with HC, respectively (Figure 4A and Supplemental Data File 1). Among these, 212 were shared between PBO-A and PBO-NA, with 35 unique to PBO-A and 41 unique to PBO-NA. Similarly, 262 and 217 autoantigens were significantly associated with RNF-A or RNF-NA compared with HCs, with 171 shared, 91 unique to RNF-A, and 46 unique to RNF-NA (Figure 4B and Supplemental Data File 1).

Overall, 100 autoantigens were common across all 4 CIS subgroups (Figure 4C). Twenty-six autoantigens were prevalent in patients with active disease (PBO-A and RNF-A), and 11 were prevalent in patients without active disease (PBO-NA and RNF-NA) (Figure 4C). These findings suggest the Antigenome Platform can detect autoantibodies enriched in patients with CIS and distinguish those with active disease.

### Protein networks of autoantibody targets associated with disease activity.

We utilized the STRING database to examine the CIS subgroup–associated autoantigens for commonalities or insight into the CIS/MS disease process. We were specifically interested in the 67 and 53 autoantigens uniquely associated with PBO-A or PBO-NA, respectively, and the 91 and 46 autoantigens uniquely associated with RNF-A or RNF-NA, respectively. For simplicity and to avoid investigating autoantibody targets that may be rare in a subgroup, we investigated the autoantigens selected by at least10% of patients within the subgroup. Thus, we investigated 35, 41, 37, and 21 autoantigens for PBO-A, PBO-NA, RNF-A, and RNF-NA, respectively (Figure 4, A and B).

For PBO-A, 12 of 35 antigens (34%) were involved in interactions; CDH2 was a hub antigen with connections to PTPRS, PXN, ARHGAP32, and FGFR1 (Figure 4D). For PBO-NA, 19 of 41 antigens (46%) interacted with each other; HSP90AB1 was a hub antigen with connections to 7 autoantigens (Figure 4E).

For RNF-A, 18 of 37 autoantigens (49%) were involved in interactions with each other. GRIA1 was a hub antigen with connections to GRIN1, ARHGAP32, and HSPA5 (Figure 4F). For RNF-NA, 4 of 21 autoantigens (27%) interact in pairs, with interactions occurring between COG7/YKT6 and CBX6/GATAD2B (Figure 4G). STRING networks showing only physical interactions are also reported (Supplemental Figure 4, B–E).

### Subcellular locations of autoantibody targets associated with disease activity.

To further explore features in the CIS-associated autoantigen targets, we used the SubCellBarcode database to identify their subcellular locations. For PBO-A, subcellular and subcompartment location data are available for 27 of 35 and 10 of 35 autoantigens, respectively, (Figure 4H and Supplemental Data File 4). PBO-A–associated autoantigens are enriched in cytosolic, mitochondria matrix, nucleosol, endoplasmic reticulum, and ribosome subcompartments.

For PBO-NA, subcellular and subcompartment location data are available for 32 of 41 and 17 of 41 autoantigens, respectively (Figure 4I and Supplemental Data File 4). PBO-NA–associated autoantigens are broadly distributed throughout the cell.

For RNF-A, subcellular and subcompartment location data are available for 31 of 37 and 18 of 37 autoantigens, respectively (Figure 4J and Supplemental Data File 4). RNF-A–associated autoantigens are enriched in the cytosol. For RNF-NA, subcellular and subcompartment location data is available for 13 of 21 and 8 of 21 autoantigens, respectively (Figure 4K and Supplemental Data File 4). RNF-NA–associated autoantigens are enriched in the cytosol and the mitochondria, specifically in the mitochondria matrix.

### Functional enrichments of autoantibody targets associated with disease activity or lack of disease activity.

Functional enrichments are reported using the STRING database (Supplemental Data File 5). The 35 investigated PBO-A autoantigens are enriched for phosphoproteins, and the 41 PBO-NA autoantigens are enriched for proteins involved in chromatin organization and acetylation. The 37 RNF-A autoantigens are enriched for phosphoproteins, chromatin regulators, protein-domain-specific binding, and endosome. The investigated RNF-NA–enriched autoantigens did not have enriched GO-Terms. These results suggest connections among some autoantigens that are physical and functional, which may provide clues to the specific targeting of these antigens.

### Predictive value of CIS autoantibody targets in the absence of therapeutic intervention.

We next evaluated whether a group of autoantigen targets predicts the occurrence of new MRI activity fulfilling either space and/or time criteria or a new clinical attack during the 2-year REFLEX trial in the absence of therapy. For model building, we used PBO-A and PBO-NA samples (*n* = 48 total) at month 0. While a combined 301 autoantigens were selected by PBO-A and PBO-NA at significance level of *q* less-than 0.2 and representation level of greater-than 1% of patients, a more stringent significance level (q < 0.05) and representation level (> 30% of patients) were utilized for predictive modeling to improve model stability by lessening the number of predictors. After this filtering, 31 autoantigens were included in model building. Disease activity status served as the response value (Y), while autoantigen sequencing counts served as predictors (X).

We built a model using Generalized Regression least absolute shrinkage and selection operator (LASSO), a regularization and variable selection method ideal for circumstances in which the number of samples (*n* = 48) is not much larger than the number of predictors (*P* = 31) (40, 41). We used nested cross validation (*K* = 4, *L* = 5) since this method incorporates validating and testing the trained model, optimizes hyperparameters to reduce overfitting, and produces unbiased and robust results irrespective of sample size (42). Briefly, nested cross-validation splits the data into a series of training, validation, and test sets. The model is developed by fitting to each training set, and then the hyperparameter conditions are optimized using the validation sets. The model is further optimized by continuing to iterate over training and validation sets (42). The success of the model is assessed using the series of test sets. The AUC gauges the performance of the model, which measures accuracy in classifying a sample as active or nonactive on a scale from 0 (completely inaccurate) to 1 (completely accurate).

**Figure 5:**
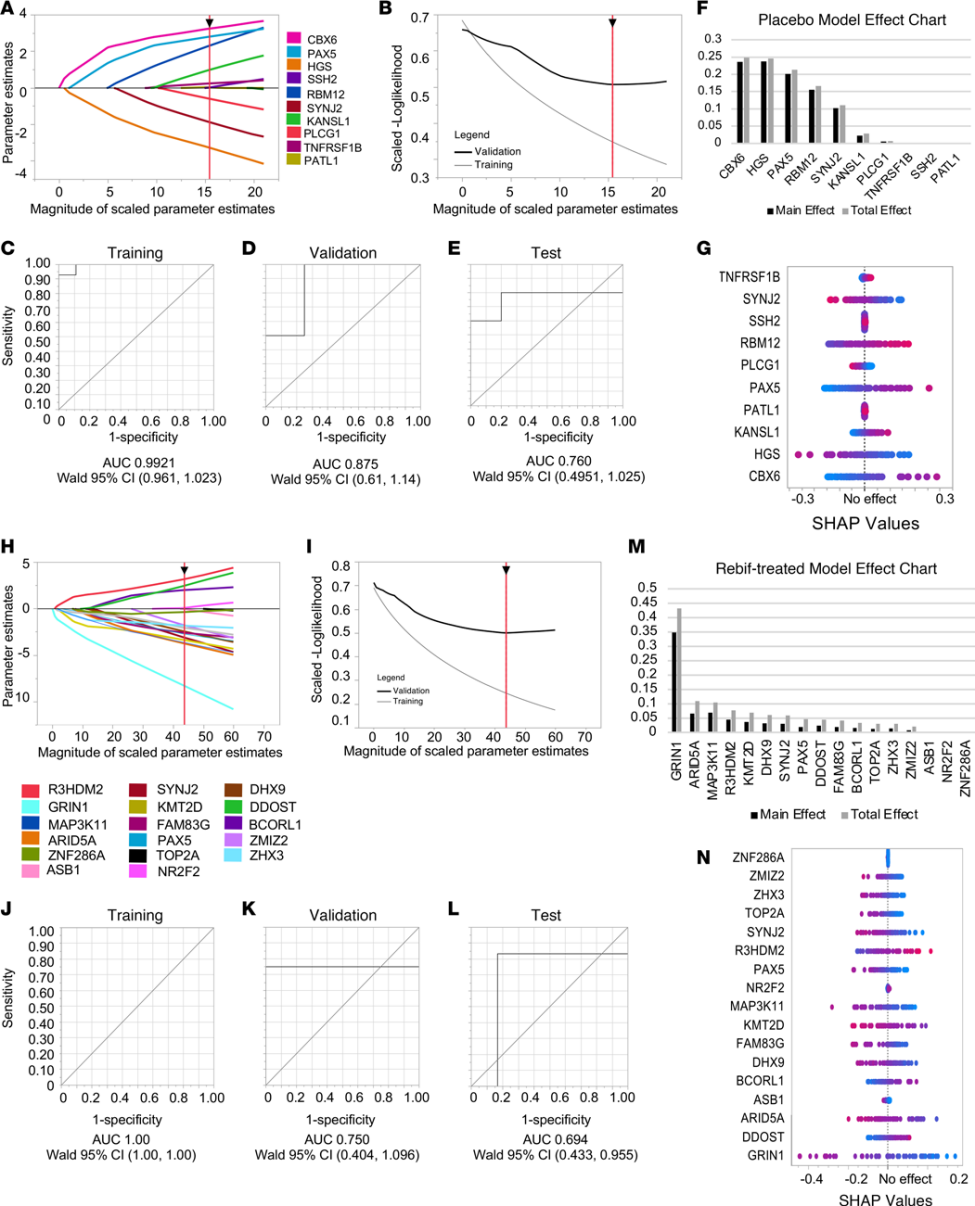
Autoantigen selection by CIS antibodies may predict disease activity and therapeutic response. (**A**–**G**) LASSO model parameters for predicting disease activity in the absence of therapeutic intervention using REFLEX placebo samples. (**A**) The Solution Path Plot displays values of the estimated parameters, where each curve represents a predictive term in the model. (**B**) The Validation Plot includes a curve for both the training and validation sets at various magnitudes of scaled parameter estimates. In each plot (**A** and **B**), the *x*-axis represents the *l*1 norm, and the vertical red line represents the value of the *l*1 norm for the best and chosen solution. (**C**–**E**) The ROC curve for the (**C**) training, (**D**) validation, and (**E**) test samples and the associated AUC values. (**F**) Effects chart. “Main Effect” shows the relative contribution of the predictor to the model alone, and “Total Effect” shows the relative contribution of the predictor when other predictors are also taken into account. (**G**) Plot of Shapley values where each dot represents a patient sample in the model. The color of the dot represents their raw value. The *x*-axis represents the effect of the sample, where a negative effect indicates a contribution to the PBO-A outcome and a positive effect indicates a contribution to the PBO-NA outcome. (**H**–**L**) LASSO model parameters for predicting response to IFN-β-1a therapy; panels were constructed as described for **A**–**G** above. (**H**) Solution Path Plot, (**I**) The Validation Plot. (**J**–**L**) ROC curves for the (**J**) training, (**K**) validation, and (**L**) test samples and their AUC values. (**M**) Effects chart. (**N**) Plot of Shapley values, where a negative effect indicates a contribution to the RNF-A outcome and a positive effect indicates a contribution to the RNF-NA outcome.

We show performance metrics for the final constructed model (Figure 5, A–G), which show a training AUC = 0.9921, validation AUC = 0.8750, and test AUC = 0.7600 (Figure 5, C–E). The “Parameter Estimate” chart values indicate whether an antigen is predictive for disease activity (negative parameter estimate) or lack of disease activity (positive parameter estimate) (Figure 5A). LASSO excludes nonpredictive antigens; this model excludes 21 predictors and retains 10 (Figure 5A).

The effects chart (Figure 5F) and SHAP (43) analysis (Figure 5G) indicate each predictor’s contribution to the model. “Total Effect” is an index based on the relative contribution of the predictor alone and in combination with other factors (Figure 5F). The top 4 antigens, CBX6, HGS, PAX5, and RBM12 contribute approximately 96% of the total effect in the model (Figure 5F), which is reflected in the SHAP analysis (Figure 5G). This model proposes 10 autoantigens for predicting active disease in untreated patients at baseline. Provided the small sample sizes used may incline the model to overfit, we recommend the predictive success of these 10 autoantigens be tested across larger and more diverse population studies.

### Autoantibody targets predict active disease in the Rebif-treated subgroup.

Since Rebif treatment is effective in only some patients with CIS, we also questioned whether any group of autoantigens serves as predictive biomarkers for Rebif IFN-β-1a response. We built a predictive model based on RNF-A or RNF-NA enriched autoantibody targets compared with HCs at baseline (Figure 4, H–N). A LASSO model was built as described above. After filtering for significance level *q* < 0.05 and representation of > 30% of patients, 27 autoantigens were included in model building. This model retained 17 predictors and excluded 10 (Figure 5, H and I). The final model has a training AUC = 1.000, validation AUC = 0.7500, and test AUC = 0.6944 (Figure 5, J–L).

The effects chart (Figure 5M) and SHAP analysis (Figure 5N) indicate each predictor’s contribution to the model. The top antigen (GRIN1) is a brain-enriched surface protein that is predictive for active disease and contributes approximately 37% of the total effect in the model (Figure 5M). The predictive value is also reflected in the dot plot of SHAP analysis (Figure 5N). Another active disease-predictive antigen, DHX9, is an RNA helicase and a known autoantibody target in the autoimmune disease lupus erythematosus (44). This model suggests 17 candidates for predicting therapeutic response to IFN-β-1a. Because the small sample sizes used may incline the model to overfit, we recommend the predictive success of these 17 autoantigens be tested across larger and more diverse population studies.

### Modulation of autoantibody production with IFN-β-1a treatment.

Since IFN-β-1a is known to modulate B cell activity, we analyzed whether the selection of autoantigens within the 4 CIS subgroups changed over time. There were no significant changes in specific autoantibodies observed over time in any subgroup based on using Wilcoxon Each Pair for statistical comparison at *q* value under 0.2. Changes in autoantibody specificities may have occurred; however, the changes may have been too small or too variable among patients to be considered significant. In our study, individual-specific variations are reflected in scatterplot R^2^ values for linear line fits (Table 5 and Supplemental Data File 6). These findings suggest that while some changes in autoantibody specificities may occur on an individual basis, IFN-β-1a treatment does not appear to modulate autoantibody production on a large-scale or in a generalized manner at the time points we assessed.

### Identification of serum autoantibodies via ELISA.

**Figure 6:**
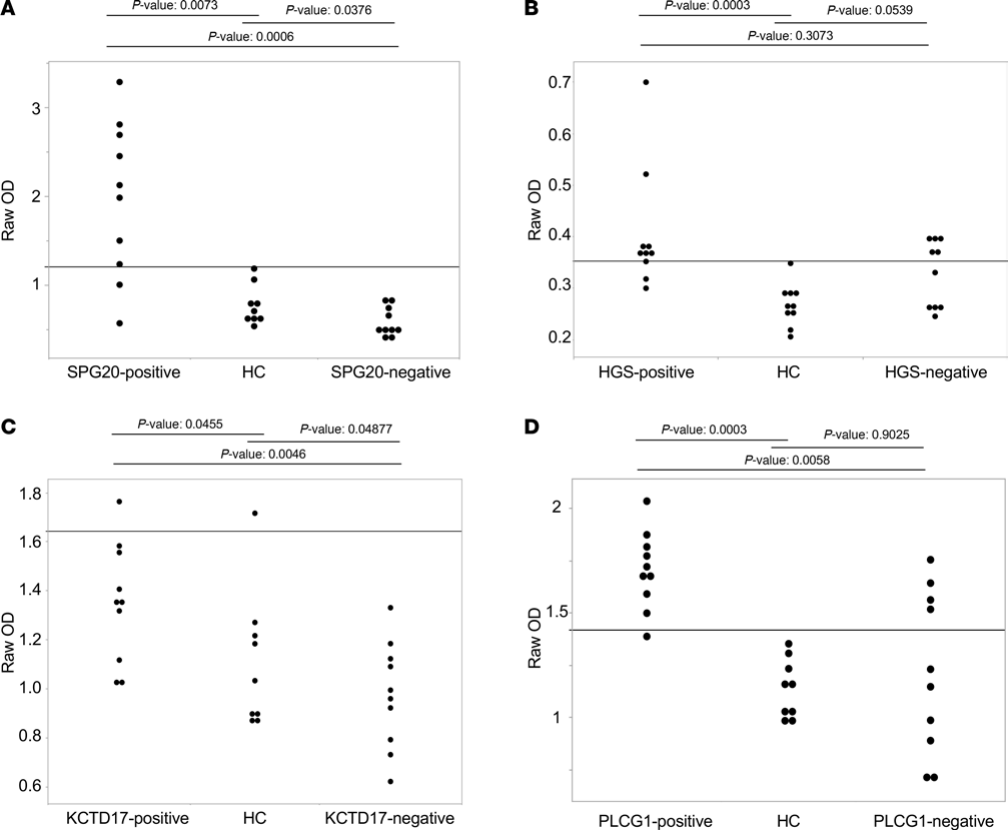
Validation of autoantibody targets via ELISA. (**A**–**D**) ELISA results assessing the presence of serum antibodies recognizing (**A**) SPG20, (**B**) HGS, (**C**) KCTD17, and (**D**) PLCG1 in patients with CIS found to be antibody-positive (*n* = 10), HC samples found to be antibody-negative (*n* = 10), or patients with CIS found to be antibody-negative (*n* = 10), via the Antigenome Platform. Kruskal-Wallis Rank Sums Test was used for statistical comparison. Black line indicates the value 2 SDs above the mean of HC samples. Outliers were detected by Robust Fit Cauchy method and removed from analysis.

We tested the potential of 4 Antigenome-identified CIS autoantibody targets to be used for clinical application via ELISA (Figure 6). Antibodies toward SPG20, HGS, KCTD17, and PLCG1 were chosen for analysis based on the commercial availability of eukaryotic-expressed purified proteins. In each case, serum antibody reactivity in the antibody-positive patients with CIS was significantly greater than that of HCs (*P* value < 0.05), showing agreement between ELISAs and the Antigenome Platform. Differences in reactivity was observed on an individual serum basis, which may be due to differences in assay sensitivity, conformation presentation allowed by each method, or epitope availability. Of the 4 autoantibodies assessed, anti-SPG20 and anti-PLCG1 were the most readily measured by ELISA and, thus, best suited for pursuing in a clinical application. Antibodies targeting HGS and KCTD17 may necessitate more comprehensive assay refinement to achieve optimal detection, which could suggest their presence at lower serum concentrations or highlight the need for optimizing the protein fragment(s) used for detection.

## Discussion

Using an original autoantigen discovery platform, we identified an array of autoantibody targets in patients with CIS and MS that may reflect underlying immune mechanisms and contribute to disease pathogenesis. The Antigenome Platform was designed to reveal the complexity of autoantigenic epitopes recognized by disease-associated antibodies by expressing extensive overlapping protein fragments of approximately 18,000 in-frame genes from 4 cell sources. Using this technology and serum samples from the REFLEX clinical trial, we identified autoantigens associated with CIS/MS disease, disease activity, and therapeutic response. Through analysis of 2 randomly partitioned groups of patients with CIS, we identified 335 reproducibly selected autoantibody targets that were enriched over that of HCs; 166 of these were selectively bound by sera of at least 10% of patients with CIS. We further investigated characteristics of these autoantigens that may reveal the basis of their targeting in CIS/MS; these findings revealed enriched autoantigen expression in brain and other tissues, shared subcellular locations, enriched GO-terms, shared structural features, and protein-protein interactions.

Several identified autoantigens interact with each other, with PLCG1 being a key hub antigen. PLCG1, an intracellular protein highly expressed in brain tissue (45), is linked to MS and other neurological disorders like Alzheimer’s, Huntington’s, and epilepsy (46). It is also a known autoantibody marker in non-Hodgkin’s lymphoma (47).

Another hub antigen is YWHAE, which is a member of the 14-3-3 protein family. Proteins of this family are found in the CSF of patients with MS and are suggested to be a marker of disease (48); however, autoantibody targeting of such proteins has not been previously reported. YWHAE is highly enriched in motor neurons in the brainstem and spinal cord in addition to the cerebellum and certain cerebral areas. Upregulation of YWHAE has been observed in various neurological disorders (48).

While most discovered autoantigens are ubiquitously expressed, 6% have enriched or enhanced brain expression, including 2 brain-enhanced surface antigens, GRIN1 and GRIA1, as shown in Tables 1 and 2. GRIN1 (also called NMDAR), is a glutamate receptor and has been previously identified as an MS autoantigen (32–37). While brain-enhanced autoantigens may be indicative of a secondary immune response occurring in the CNS due to the excess of cell damage and debris, brain-enhanced cell-surface autoantigens may also facilitate damage in the CNS; autoantibodies targeting other neural glutamate receptors cause neuronal cell damage both through excitotoxicity and complement fixation (49). Since B cell autoantigens are likely also recognized by T cells, damage may occur through autoantibodies, B cells, or T cell mechanisms. Thus, GRIA1 and GRIN1 are candidates for a role in MS pathology, which should be investigated in future studies.

Of interest, we found that approximately 10% of CIS-associated autoantigens have enhanced expression in the skeletal muscle, including FLII and SVIL, both members of the villin family of actin-binding membrane proteins (50). While the significance of this finding is unclear, patients with MS often experience muscle-related clinical manifestations such as reduced muscle mass, changes in tissue composition, and muscle weakness (51–53). These symptoms can appear early in CIS and may help predict further demyelinating events. Recent evidence suggests that changes in the skeletal muscle function directly contribute to MS disability progression (54); autoantibodies targeting muscle-enhanced proteins may reflect such skeletal muscle damage.

GSEA shown in Figure 3 and Supplemental Figure 3 indicate that autoantibodies in CIS favor protein targets with more hydrophilicity and β turns, which mirrors previously reported autoantibody biases (55). As shown in Tables 3 and 4, we further identified enrichments in protein function and complexes through GO-terms, including an enrichment for members of the NuRD complex, which includes CHD4. CHD4 is a known target of autoantibodies in the inflammatory myopathy called dermatomyositis, where anti-CHD4 positivity is associated with more severe muscle disease (56, 57).

Go-term analysis also revealed an enrichment of proteins involved in cell junctions, which includes CDH2, HSPA5, CD99L2, and LSR, among others. CDH2 is a transmembrane glycoprotein that is highly expressed in the CNS, and deletion of CDH2 increases junctional endothelial permeability in the brain (58). Disruption of CDH2 function through blocking the extracellular interaction domain also triggers massive apoptosis of ependymal cells and denudation of brain ventricular walls (59, 60). Thus, the autoimmune targeting of CDH2 may play a role in blood-brain barrier disruption.

HSPA5 is a heat shock protein that, while primarily located in the endoplasmic reticulum, can translocate to the cell surface in stressed cells where it binds to extracellular ligands (61). Cell-surface HSPA5 is known to induce autoantibody production is various diseases (62–67), including other neurodegenerative autoimmune diseases where autoantibodies targeting HSPA5 have been implicated in demyelination, astrocytopathy, and blood-brain barrier disruption (68).

In addition to investigating autoantigens enriched in the general CIS population, we also found that autoantibody specificities mark those with active disease among patients with CIS and potentially mark short-term response to IFN-β-1a, as shown in Figures 4 and 5. Moreover, our results align with prior demonstrations of autoantibody specificities stratifying clinical variants that differ from classic MS in their response to therapy and disease course (69, 70).

Data presented in Figure 4 show 35 and 41 autoantibody targets enriched in PBO-A and PBO-NA, respectively, which includes the previously discussed cadherin protein CDH2, enriched in PBO-A. Additionally shown are 37 and 21 autoantibody targets enriched in RNF-A and RNF-NA, respectively, which includes the previously discussed cell junction proteins GRIA1, GRIN1, ARHGAP32, and HSPA5, enriched in RNF-A.

The data presented in Figure 5 show that the expression of antibodies to 10 and 17 autoantigens predicts the occurrence of new MRI or clinical activity in placebo or patients treated with IFN-β-1a, respectively, during the 2-year REFLEX trial. Expression of antibodies to GRIN1 contributed great predictive value (approximately 37% of the total effect in the model) for patients with active disease while being treated with IFN-β-1a. In the absence of therapeutic intervention, expression of antibodies to a group of 10 autoantigens was predictive for disease activity status, and binding of antibodies to 4 autoantigens, CBX6, HGS, PAX5, and RBM12, contributed the vast majority (approximately 96%) of the predictive value. A large-scale assessment of the predictive value of these autoantibodies using additional CIS samples and commonly available methodologies, such as an ELISA, will specify the extent of clinical utility that assessing these autoantibodies provides.

Even though we ascertained autoantigen targets associated with a response to IFN-β-1a therapy, we did not find evidence for changes in autoantibody specificities in a consistent and statistically significant manner in treated patients, as shown in Table 5. It should be considered that changes in autoantibody profiles may be observed at different time points in the treatment than the time points assessed in this study; markers of this kind may also be specific for an individual. It also remains possible that our methodology and data analysis strategy may not sensitively reflect small or individual changes in autoantibody titer. Nonetheless, a recent study on autoantibody expression in MS over a 6-year period also did not find changes in MS autoantibody expression over time (71). Furthermore, studies using conventional serologic assays have not found changes in autoantibodies with IFN-β-1a treatment (72–77); however, other studies do report changes (78–83). This lack of agreement among studies could be due to differences in patient populations tested or the specific autoantibodies assessed (84).

Our findings are also consistent with observations that autoantibodies in patient sera bind brain cells (10, 85); as indicated in Tables 1 and 2, our data show that at least 10% of patients with CIS express antibodies toward the neural cell-surface proteins GRIN1, GRIA1, and SORT1 and the neural intracellular antigens KIF1C, SYNJ2, EXPH5, KCTD17, CBX6, and TRAK2. Tables 3 and 4 also reports enrichment of autoantibodies targeting nucleic-acid binding proteins; the expression of autoantibodies to DNA/RNA and DNA/RNA-binding proteins is a common feature of autoimmune diseases. This result is consistent with previous demonstrations of sera from patients with MS being positive for antinuclear antibodies (39).

Tables 3 and 4 also shows that most autoantigens are located intracellularly, which is consistent with previous reports showing that oligoclonal bands can target ubiquitous intracellular antigens, possibly released as cellular debris (86). In view of current models for the pathophysiology of MS, antibodies toward intracellular targets are unlikely to initiate demyelination and inflammation; rather, such autoantibody responses could arise subsequent to cell damage and contribute to disease by amplifying ongoing inflammation, promoting or inhibiting the removal of damaged myelin and cells, impairing recovery, or facilitating potential epitope spreading.

Studies in other diseases have demonstrated that only some discovered biomarkers are convertible to clinical application while others serve research purposes only (87). Differences in methodology can lead to differences in detection and can pose challenges for standardizing clinical assays. For example, in our study, while the Antigenome Platform allows for detection of antibody reactivity to internal or hidden epitopes that may be revealed during disease processes, the ELISAs used in this study assessed antibody binding to intact proteins. The proteins assessed in the Antigenome Platform are also phage produced, whereas the ELISAs assessed binding to mammalian cell–produced proteins; this could lead to differences in antibody detection due to posttranslational modifications or differences in the availability of chaperone proteins. The data analysis conducted in this study evaluated autoantibody binding to entire proteins. However, the platform enables investigation into specific protein fragments targeted by autoantibodies, which could yield valuable insights. Such an analysis may guide the selection of optimal autoantibody targets that are more readily detectable by ELISA, especially by prioritizing targets with surface-level epitopes. Additionally, while the Antigenome Platform theoretically allows for identification of either conformational or linear epitopes, we are unable to determine by this technology alone which type of epitope is bound by an antibody, which may affect the success of a clinical test. Nevertheless, improvements in clinical assay methodology will continue to broaden the spectrum of targets assessed clinically. Based on ELISA assays, we believe that some of the autoantibodies identified in this study will be good candidates for detection for clinical purposes via commonly used laboratory format assays. Protocols for clinical detection of Antigenome Platform–discovered autoantibodies should be carefully designed and optimized, which should include selection of relevant protein fragment (or fragments) for detection and setting meaningful bounds of positivity.

A limitation of the study relates to the classification of patients. The REFLEX study assessed conversion from CIS to McDonald MS as ascertained by the 2005 McDonald criteria (6), which have been revised in 2010 (88) and 2017 (89). Conversion to MS under the McDonald 2005 criteria during the REFLEX trial necessitated either (a) new MRI activity fulfilling either space and/or time criteria or (b) a new clinical attack (6). Thus, due to changing MS diagnostic criteria, we considered conversion during the trial to designate any new clinical or MRI lesion (disease activity) and lack of conversion to denote lack of disease activity. Since study criteria did not exactly match McDonald 2017 criteria, one subject in the “no activity” group had a new small MRI lesion noted but was considered inactive. Future prospective studies using samples classified according to the 2017 McDonald criteria will improve sensitivity and validate candidate targets.

Furthermore, this study focused on autoantibody assessments exclusively in patient sera. While identifying autoantibodies in the CSF would be informative, the low concentration of IgG in the CSF makes sera better suited for discovery analysis. Furthermore, there is evidence that autoantibodies found in patient sera may reflect the autoantibodies present in the CSF; for example, tracing the clonal maturation of B cells in MS indicates a bidirectional exchange of B cells between blood and CSF and suggests that clonally related B cells exist in the meningeal lymphoid tissue, CSF, brain lesions, blood, and peripheral lymphoid tissues (85). Future studies should confirm the presence of the identified autoantibodies in patient CSF.

In conclusion, this study describes the Antigenome Platform technology and employs it to identify an array of autoantibody targets reproducibly found in patients with CIS/MS. The data suggest that some of these targets may contribute to MS disease pathology and/or serve as biomarkers for diagnosis, prognosis, or theragnosis. Thus, this study provides profiles of autoantibody targets for continued analysis; future studies should detail the extent of clinical utility these markers will serve and the contribution of these autoantibodies in disease pathogenesis.

## Methods

### Sex as a biological variable.

Serum samples from males and females were included, and sex-matching was considered in selecting samples for each of the 4 CIS study subgroups.

### Statistics.

All statistical comparisons were made with Kruskal-Wallis Rank Sums Test or Wilcoxon paired test (longitudinal analysis), which were implemented with JMP Pro Software. False Discovery Rate (FDR) correction was applied to the resulting *P* values (using a JMP Pro add-in). Antigens with a *P* value < 0.05 and a *q* value < 0.2 were considered statistically different between comparison groups.

### Study approval.

The purpose of this study was to design a stringent and expansive autoantigen discovery system and apply that system for the study of MS. Toward this goal, we designed and utilized a 2-plasmid–based cDNA selection system for stringent selection of in-frame ORFs, phage-display technology, a rigid serum antibody-selection process, next generation sequencing, and a robust bioinformatics and data analysis pipeline. This technology was applied to studying autoantibody targets in MS using human serum samples from patients with CIS and donors who were HCs.

Transcripts from HEp-2 cells, PBMCs, astrocytes, and white brain matter (WBM) were used to make cDNA libraries. HEp-2 cells were obtained commercially, PBMCs were collected from healthy donors at Duke University under IRB-approved protocols, and astrocytes and WBM were a contribution from the University of Colorado, Denver.

Human serum samples from healthy individuals were collected at Duke University. 306 CIS serum samples were obtained from EMD Serono, which were collected during the REFLEX clinical trial (14–16). Informed consent was obtained under the established IRB guidelines applicable to each serum source.

### Data availability.

All data associated with this study are present in the paper or the Supplemental Materials as well as available from the corresponding author upon request. A detailed account of all methods used in this study can be found in the Supplemental Materials. Supporting data values are available as a supplemental file.

## Author contributions

EBD and TT conceptualized the project and developed project aims. TT supervised internally. DEH, SL, ERP, and JLB provided external supervision and mentorship. EBD, EK, WZ, and TT developed the methodology. DEH and SL provided MS serum samples. JLB provided brain matter and astrocyte biomaterials. DP and TT provided HC serum samples. EBD conducted statistical, computational, and data analysis (excluding rabbit anti-sera and propensity score matching). ERP supervised bioinformatic and statistical analysis. EK analyzed rabbit anti-sera. BH performed propensity score matching. EBD and MZ created pBAD-CO, PHAGEMID-CO, human cDNA, and phage libraries. EBD and JH assayed samples with the Antigenome Platform. EK assayed rabbit anti-sera. JK was the project administrator. DEH, SL, and TT acquired funding. EBD wrote the original draft of the manuscript. EBD, DSP, and JLB wrote revisions; all authors provided commentary.

## Supplementary Material

Supplemental data

Supplemental data set 1

Supplemental data set 2

Supplemental data set 3

Supplemental data set 4

Supplemental data set 5

Supplemental data set 6

Supporting data values

## Figures and Tables

**Table 2:**
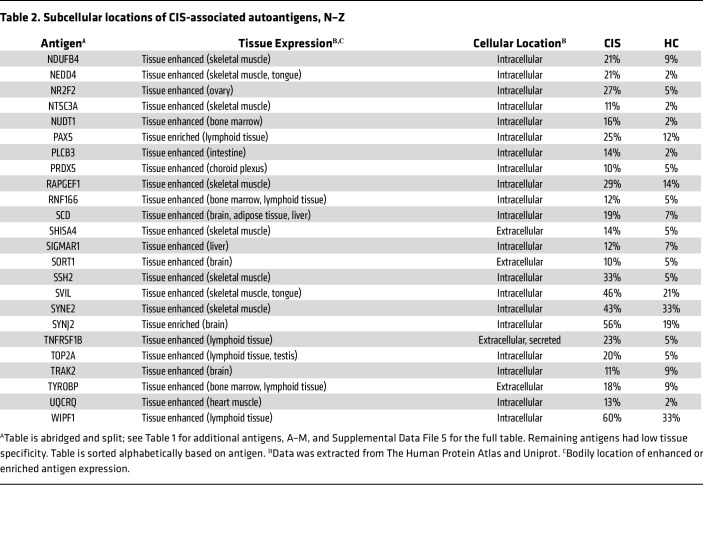
Subcellular locations of CIS-associated autoantigens, N–Z

**Table 4:**
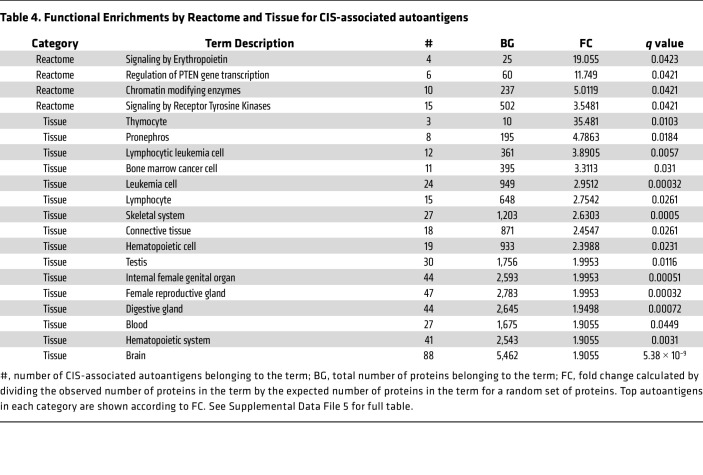
Functional Enrichments by Reactome and Tissue for CIS-associated autoantigens

**Table 1:**
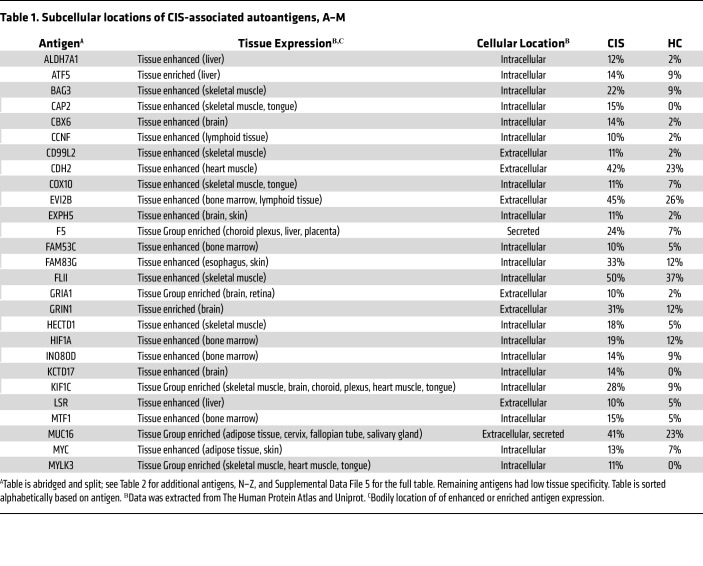
Subcellular locations of CIS-associated autoantigens, A–M

**Table 3:**
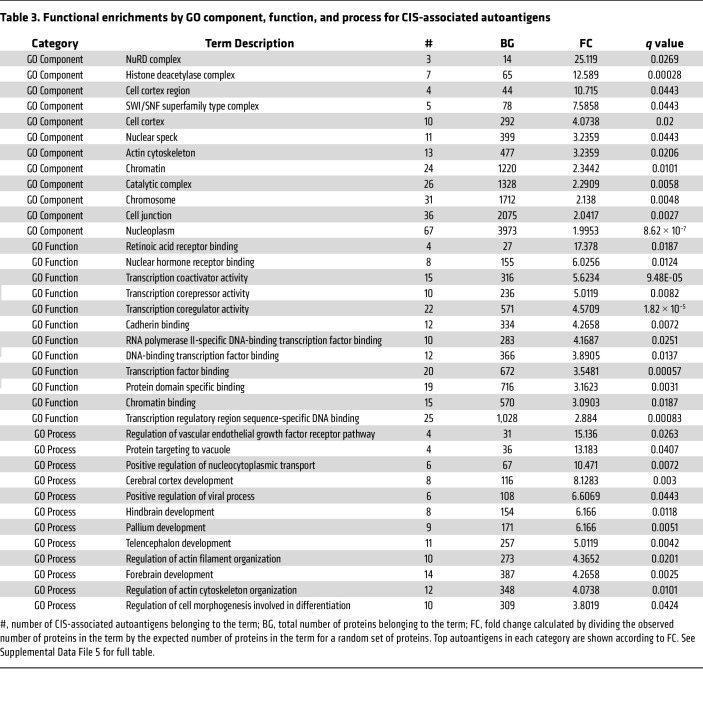
Functional enrichments by GO component, function, and process for CIS-associated autoantigens

**Table 5:**
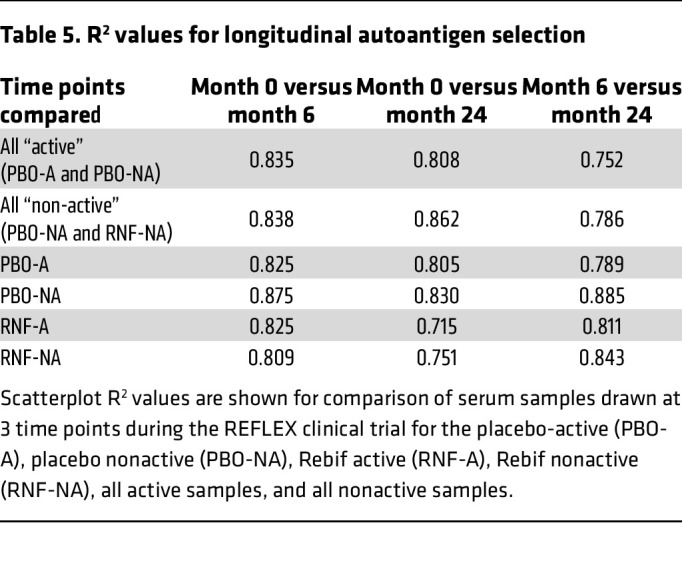
R^2^ values for longitudinal autoantigen selection

## Version 1 03/03/2025

Electronic publication
