# Supplementary material for: Patterns of autoantibody expression in multiple sclerosis identified through development of an autoantigen discovery technology
Source: J Clin Invest. 2025 Mar 3;135(5):e171948. doi: 10.1172/JCI171948 (PMC11870742; doi:10.1172/JCI171948)
Supplement: Supplemental data [file jci-135-171948-s083.pdf]

Supplemental Methods

Phage Library Construction – First Plasmid

The approach we used to construct human cDNA libraries advances work published in Biotechniques in 2004 (1), which expansively describes some of the methods described briefly herein.

RNA harvesting:

Transcripts from HEp2 cells, PBMCs, astrocytes, and white brain matter (WBM) were used to make cDNA libraries. HEp2 cells were obtained commercially, PBMCs were collected from healthy donors at Duke University under IRB approved protocols, and astrocytes and WBM were a contribution from University of Colorado Denver. Total RNA was harvested from cells using TRIzol (Invitrogen), and rRNA was depleted using RiboMinus (Thermo Scientific), both according to the manufacturer's instructions.

First-strand cDNA synthesis:

mRNA from each of the four cell types was used as a template for first cDNA strand synthesis (in four separate reactions). mRNA was combined with NOTIRAN2 primer (5' GCGGCCGCAACNNNNNNNNN 3'), water, and dNTP. NOTIRAN2 is a random primer with a known tag including a *Not* I tag and hybridizes with PCR primers for amplification in a future step. The reaction was incubated for 5 minutes at 65°C, then on ice for 1 minute. Subsequently, SSII Reverse Transcriptase (Invitrogen), SSII buffer (Invitrogen), DTT, and RNAsin Plus were added.

The mixture incubated for 10 minutes at 25°C, then 70 minutes at 42°C, then 15 minutes at 70°C. Primers were removed using AxyPrep DNA size selection beads (Corning) according to the manufacturer's instructions.

<u>Second-strand cDNA synthesis:</u>

The second cDNA strand was synthesized using the product from first-strand cDNA synthesis. To the first-strand purified reaction, we added NCOIRAN2 primer (5' TGGCCGCCGAGAACNNNNNNNNNN 3'), NEB Buffer2, and dNTP. NCOIRAN2 is a random primer with a known tag for cloning. The reaction incubated for 4 minutes at 95°C, then 1 minute on ice. Klenow exo-minus (NEB) was added, and the reaction was incubated at 23°C for 10 minutes, then at 37°C for 1 hour. NCOIRAN2 primers were removed by AxyPrep beads.

<u>PCR amplification of cDNA:</u>

The constructed cDNA from each of the four cell types was used as a template for PCR using *Not* I-Gibson (3' TTTTACTTTCACCAGCGTTTCTGGGTGAGCTGCAGCGGCCGCAAC 5') and *Nco* I-Gibson primers (3' GCTGGTGGTGCCGTTCTATAGCCATAGCACCATGGCCGCCGAGAAC 5'), which hybridize with the cDNA sequence tags. These primers introduce an *Nco* I site and tags for cloning. PrimeStar Premix (Takara) was added to the reaction. A thermocycler was used for the PCR reaction with the following parameters:

95°C, 2 minutes

____

94°C, 20 sec

62°C, 10 seconds 15 cycles

72°C, 45 seconds

____

72°C, 45 seconds

The amplified DNA was size selected to remove primers and small fragments using AxyPrep beads (Corning).

Selection of cDNA inserts enriched for ORFs

The vector pBAD Select was used for stringent selection of in-frame cDNA ORFs. First, we modified the pBAD Select plasmid for codon optimization (CO) and renamed it pBAD-CO. pBAD-CO contains *Nco* I and *Not* I sites in the MCS, followed by an ampicillin resistance gene, all under control of the araBAD inducible promoter. In this design, when a cDNA fragment is inserted between the *Nco* I and *Not* I sites, ampicillin resistance is only conferred when the inserted DNA is an ORF lacking any stop codons.

pBAD-CO plasmid was digested with *Not* I-HF (NEB) and *Nco* I-HF (NEB) and gel purified to remove the stuffer. The PCR-amplified human cDNA fragments (derived from each of the four cell types) were cloned into the open pBAD Select plasmid using Gibson Assembly (NEB) cloning

methods according to the manufacturer's instructions.

Phi29 amplification of pBAD-CO library

The four ligated plasmid libraries were combined with WGA N7 primer (3' NNNNNN*N* 5'; * denotes phosphorothioate modifications) and dNTP in Phi29 buffer. The reaction was incubated for 3 minutes at 70°C and cooled on ice. Subsequently, the DNA polymerase Phi29 was added (Lucigen) for whole plasmid replication according to the manufacturer's instructions. The reaction incubated for 16 hours at 30°C, then 10 minutes at 65°C.

The libraries were digested with *Not* I-HF (NEB) to resolve any concatenated product and re-ligated using T4 DNA ligase (NEB). The resulting pBAD-CO plasmid library product was column purified using a micro concentrator kit (Zymo Research).

pBAD-CO Transformation

E. cloni 10G Elite electrocompetent bacteria (Lucigen) was modified to express pRARE2, and sent to Intact Genomics (www.intactgenomics.com) to be made highly competent. pRARE2 plasmid expresses additional tRNAs to combat codon bias (90). Subsequently, the bacteria was transformed with each of the four pBAD-CO libraries through electroporation. The bacteria recovered in SOC media for 30 minutes at 37°C before expanding into 2YT media with 0.2% arabinose and 30 ug/uL carbenicillin. Bacteria were grown at 30°C until 0.4 OD^{600}). On average, the transformational efficiency was 4.9 x 10^8 colonies. Plasmid DNA was prepared from the transformed bacteria via

midi prep kit (Zymo Research).

Phagemid Library Construction – Second Plasmid

Preparing the second plasmid (PHAGEMID-CO) for cloning

For phage library construction, we used the pSEX81 phagemid (available through Progen), which we modified for codon optimization (CO) (called PHAGEMID-CO). After selection for in-frame ORFs in the pBAD-CO vector, DNA inserts were cloned from pBAD-CO into PHAGEMID-CO using Gibson Assembly. PHAGEMID-CO contains a leader sequence, followed by a MCS containing *Nco I* and *Not* I, followed by pIII phage fusion protein. Thereby, if any cDNA inserts contain stop codons, the pIII phage protein will not be produced, inhibiting infectious phage assembly. Thus, phage production is a second selection step.

Preparing cDNA inserts for cloning into PHAGEMID-CO

cDNA inserts were PCR amplified from all four pBAD-CO libraries using Hybridization-Part-2-*Not* I primer: and Hybridization-Part-2-*Nco* I primers.

Hybridization-Part-2- *Not* I primer	3’ TAAGCAGGATTCAACAGTTTCAGCGCGGATATCTTTGGAACCAGCGGCCGCAAC 5’
Hybridization-Part-2-*Nco* I	3’CCGCTGGCTTGCTGCTGCTGGCAGCTCAGCCGGCCATGGCCGCCGAGAAC 5’

These primers hybridize with the pBAD-CO sequencing flanking the human cDNA inserts and

add flanking sequences that will hybridize with PHAGEMID-CO. PrimeStar premix (Takara) was added to the reaction. The following PCR program was used:

95°C, 2 minutes

94°C, 20 seconds

47°C, 10 seconds 12 cycles

72°C, 45 seconds

72°C, 45 seconds

After PCR, 1 ul of DpnI (New England Biolabs) was added to the reaction and incubated for 1 hour at 37°C, followed by size selected with AxyPrep beads to remove parent plasmids and primers. The cDNA inserts were then cloned into PHAGEMID-CO using Gibson Assembly (NEB).

Phi29 amplification of PHAGEMID-CO library

The phagemid library was combined with WGA N7 primer (3' NNNNNN*N* 5'; * denotes phosphorothioate modifications) and dNTP in Phi29 buffer. The reaction incubated for 3 minutes at 70°C. Subsequently, the DNA polymerase Phi29 was added (Lucigen) for whole plasmid replication. The reaction incubated for 16 hours at 30°C, then for 10 minutes at 65°C.

Transformation of PHAGEMID-CO Library

SS320 electrocompetent bacteria (Lucigen) was modified to express pRARE2, as described above, and transformed with the PHAGEMID-CO library by electroporation. Bacteria recovered in SOC media for 30 minutes at 37°C before expanding into 2YT with 1% glucose and 30 ug/uL carbenicillin. Glucose was used to eliminate cDNA insert-derived competitive growth advantages by inhibiting protein production. Bacteria grew at 30°C until 0.4 OD^{600}. Plasmid DNA was prepared from the transformed bacteria via midi prep (Zymo Research).

Producing Phage

Phage particles were produced from the phagemid-transformed bacteria and purified as previously described (1). The infectious phage titer was determined by infecting pRARE2+ SS320 bacteria with phage and counting bacteria colonies. Individual bacteria clones were picked for plasmid preparation (Qiagen Plasmid Mini-Prep Kit), which were Sanger sequenced (Genewiz) to assess the percentage of in-frame ORFs encoding proteins. Phage with DNA inserts less than 40 bp, or those with deleted insert-flanking sequences were eliminated from analysis as they are purged throughout the assay through bead-based size selection.

Sequencing Libraries

Next-generation Sequencing HiSeq (Genewiz) informed the diversity of each phage library. cDNA inserts were PCR amplified from phagemids isolated from the phage libraries (Qiagen mini-prep

kit). An Index Primer (5’ CAAGCAGAAGACGGCATACGAGATXXXXXXGTGACTGGAGTTCAGACGTGTGCTCT TCCGATCT 3’, where X represents individual index sequences for barcoding each sample) and a Universal primer (5'AATGATACGGCGACCACCGAGATCTACACTCTTTCCCTACACGACGCTCTTCCGATC TCCATGGCCGCCGAGAAC 3’) were used for PCR. Both primers incorporate sequence needed for next-generation sequencing. All four libraries were pooled equally based on DNA concentration and sequenced on one Illumina HiSeq lane.

Serum Samples

Human serum samples from healthy individuals were collected at Duke University. The time course serum samples from Donor #1 were all collected at Duke University from a male over 50 years old with celiac disease and interstitial lung disease. Informed consent was obtained under the established IRB guidelines applicable to each serum source.

306 CIS/MS serum samples were obtained from EMD Serono, which were collected during the REFLEX clinical trial (2-4). The REFLEX study assessed conversion from CIS to McDonald MS as ascertained by the 2005 McDonald criteria (5), were revised in 2010 (6) and 2017 (1). During the REFLEX trial, conversion to MS based on the McDonald 2005 criteria required either new MRI activity meeting space and/or time criteria or a new clinical attack. To address the evolving MS diagnostic criteria, we designated patients who converted during the trial as patients with disease activity since during the REFLEX trial, conversion to MS based on the McDonald 2005 criteria required either new MRI activity meeting space and/or time criteria or a new clinical attack.

Patients who were designated as non-converting during the REFLEX trial were designated as lacking disease activity in our study. Since the study criteria didn't exactly align with the McDonald 2017 criteria, one participant in the "no activity" group had new small MRI lesions but was still considered inactive. 33% of the PBO-A cohort, 5% of the PBO-NA cohort, 19% of the RNF-A cohort, and 37% of the RNF-NA cohort would have been assigned a converting status at baseline according to the McDonald 2017 criteria.

Propensity score (PS) matching was applied for selecting the 306 MS REFLEX trial samples. Three sets of propensity scores were generated using logistic regression models to estimate the predicted probability of being in the placebo-treated, non-active subgroup vs. each of the other 3 subgroups as a function of available potential confounders: (1) age at baseline, (2) sex, (3) monofocal or multifocal presentation, (4) steroid use at first event, (5) number of gadolinium (Gad)-positive lesions in the most recent MRI data prior to baseline, and (6) number of T2 lesions in the most recent MRI data prior to baseline. For PS matching, a greedy nearest neighbor algorithm was used to find the closest 1:1 match for the placebo-treated, non-active patients in each of the other 3 patient groups. An additional 6 closest matches per subgroup were selected to reach ~100 total samples. The placebo-treated, non-active group, with the smallest sample size, was chosen to be the control group in order to retain the largest number of patients and a similar number across all subgroups. The distributions of the PS were confirmed to overlap using descriptive statistics before and after matching. To assess the balance of confounders between each pair of patient subgroups before and after matching, the standardized difference was calculated based on Cohen's d for continuous or categorical (up to two levels) variables, and Cramer's V for other categorical variables, where applicable. PS matching and balance diagnostics were

performed in R Statistical Software (7).

The 102 MS serum samples collected at Month 0 were randomly distributed between two groups, CIS – Set 1 and CIS – Set 2 with the four subgroups evenly distributed between the two groups for balance. Thus, we separated the data into the four subgroups (PBO-A, PBO-NA, RNF-A, RNF-NA), then we used the Microsoft Excel function "Rand" to randomly sort the samples, then we designated the top half of the samples into CIS – Set 1 and the bottom half of the samples into CIS – Set 2.

Rabbit anti-sera

The rabbit anti-sera purchased for Figure 2B have the following catalogue numbers: CALD1 (A304-164A), PCNA (A300-277A-T), NONO (A300-587A), ABI2 (A302-499A), UBA1 (A301-125A), ATN1 (A300-753A), ITGB1 (A303-735A), LDHB (A304-770A), DDX5 (A300-523A), MAPK9 (FL): sc-572, CAV1 (N-20): ssc-894, THRAP3 (A300-956A), SOS1 (sc-259), RAC1 (sc-217), and SHC1 (sc-288).

Serum Antibody Immunoselection of Phage Library Protein

All four phagemid libraries (deriving from HEp-2 cells, PBMCs, WBM, or astrocytes) were combined in a 1:4 ratio based on phage titer. 10^{10} phage from the combined library were incubated with 10 ul of 1:10 diluted human sera (~10 ug of IgG) at 4°C with rocking for 12 hours. 75 ul of pre-blocked Pierce Protein G Magnetic Beads (Thermo Fisher Scientific) was added and the mixture was incubated for 2 hours at 4°C with rocking. Unbound phage were washed away using KingFisher Duo Prime sample purification system (Thermo Fisher Scientific), and selected phage

were eluted from beads with trypsin. In this assay, there is a theoretical 4.5x excess of Protein G beads compared to serum IgG antibody, assuming an average serum IgG concentration of 10 mg/mL. This amount was chosen with the goal of retaining an excess of beads despite any variability in serum IgG concentration and bead binding capacity.

Eluted phage were used to infect pRARE2+ SS320 bacteria to produce a once-selected phage library. Phage were produced and purified from the infected pRARE2+ SS320 bacteria as previously described (1). The once-selected phage library was used for antibody selection once more following immunoselection procedures described above. The twice-selected phage were used to infect SS320 bacteria, and PHAGEMID-CO plasmid libraries were harvested for each sample using a miniprep kit (Qiagen). From the isolated phagemids, cDNA inserts were amplified and prepared for library sequencing as described above. Twenty-five indexed sample libraries (including an assay control and input library) were pooled equally according to DNA concentration and sequenced on a single Illumina HiSeq lane (Novogene or Genewiz).

Bioinformatics

Next-generation sequencing reads were demultiplexed by Novogene or Genewiz. Reads were trimmed using Cutadapt (version 3.2) (8) and aligned to the human genome using the STAR alignment tool (version 2.7.7a) (9). Given the evidence for the contribution of alternative splicing to the generation of untolerized epitopes and autoimmunity, reads may map to exons or introns (10). Reads were then binned by protein using HTSeq (version 0.13.5) (11). Fragment level data for Figure 1E was obtained using the Python package PySam (-m pip install pysam). Python3 was

used for processing. Modified relative log expression (RLE) was applied using the Bioconductor package DEseq2 for Fig. 2B.

Date processing

Batch correction

The Donor #1 serum sample is sequenced in every batch as an assay control. Batches were highly consistent obviating correction needs. However, some batches had overrepresentation of certain antigens across all samples in the batch. Thus, Donor #1 was used to correct these overrepresentations. The sequencing counts for Donor #1 for a batch was divided by the average sequencing counts across all Donor #1 batches, and the antigen's sequencing counts for all samples in the batch were divided by that resultant number. Since small variations in low sequencing counts would lead to large corrections, corrections were only made when the Donor #1 sample had at least 200 counts. 97% of antigens were divided by 1 and thus were not batch corrected. Therefore, batch correction was seldom needed.

Binary data transformation

Transforming sequencing selection counts into "positive" or "negative" binary form occurred by calculating a threshold of positivity for each protein, which is based on the median and the median absolute deviation of the healthy control population. (Threshold of Positivity) = [(median sequencing counts in the healthy control population) + (3 x median absolute deviation in the

healthy control population)]. A value is considered positive if its sequencing count is above this threshold and greater than 200 counts, otherwise, it is considered negative.

Volcano plots

Negative log10 of p-values (y-axis) were obtained through the Kruskal-Wallis Test. The difference of log2-transformed mean selection counts were calculated for each represented group (y-axis). JMP Pro statistical software was used for statistical analyses and graph building.

Construction of protein-protein network.

Search Tool for the Retrieval of Interacting Genes (STRING; www.string-db.org, version 11.5) was used to construct protein-protein networks. The "multiple protein" search function was used and settings were adjusted to include Full STRING network or physical subnetwork, depending on the figure, which is specified in the figure legends. MCL clustering was applied for CIS-all.

Subcellular Locations

Subcellular locations diagrams and information for charts were obtained from SubCellBarcode (subcellbarcode.org) (12), where the "Network Multi Protein Localization" tool and the A431 cell line was used.

Human Protein Atlas

Plots showing antigen RNA levels in human tissues were constructed in JMP Pro graph builder using "RNA consensus tissue gene data" downloaded from The Human Protein Atlas (www.proteinatlas.org/about/download). Annotations of antigens being "enhanced", "enriched" , or "Low Tissue Specificity" was derived from The Human Protein Atlas (www.proteinatlas.org), where their stated definitions of the terms were used (13).

GoNet

The GoNet database (www.tools.dice-database.org/GOnet) (14) was used to identify enriched biological processes or molecular function GO-Terms. A q-value threshold of < .05 and a background of "all annotation genes" were used.

Enrichment over input library

We calculated enrichment over the input library by dividing a patient's sequencing counts for autoantibody selection by the sequencing counts of the input library. These enrichment values were then used to compute mean, median, and range values for each patient subgroup. The input library values used in the calculations were an average of input library sequencing counts across all sequencing batches.

Biochemical properties

The hydrophobicity, aromaticity, isoelectric point, and fraction of amino acids in sheets and turns were calculated using Biopython (version 1.81) module Bio.SeqUtils.ProtParam for Python (version 3.8.10). The values of Chou & Fasman beta turns, Emini surface accessibility, Karplus &

Schulz flexibility, and Parker hydrophilicity across the proteins were calculated the Immune Epitope Database (IEDB) downloadable command-line tools (www.IEDB.org) (15). These values were averaged for each protein. The biochemical property values were then used for GSEA using the desktop version of "GSEAPreranked" (15, 16).

ELISA

The enzyme linked immunosorbent assays (ELISA) were performed according to standard protocols. Half area, flat-bottom 96-well microtiter plates (Corning) were used. All washing steps were performed with PBS with 1% Tween three times. All reagent volumes were 30 μl/well except blocking, which was 100 μl/well. The coating antigens used were chosen based on commercial availability of mammalian cell-produced human proteins: PLCG1 (Origene Technologies, Cat# TP316448), KCTD17 (Origene Technologies, Cat# TP306070), HGS (Origene Technologoes, Cat# TP306070), and SPG20 (Origene Technologies, Cat# TP327162). HRP-conjugated anti-human IgG heavy chain was used as a secondary antibody (Thermo Fisher Scientific, Cat# A5686250UL). Before assaying with sera, ELISAs methods were validated using commercially available antibodies for each tested protein: PLCG1 (Thermo Fisher Scientific, Cat# 28362-1-AP), HGS (VWR, Cat# 76464-170), KCTD17 (Thermo Fisher Scientific, Cat# PA568686), and SPG20 (Life Technologies Corporation, Cat# PA5106297). TMB substrate and stop solution was purchased from Abcam. 10 serum samples per group (antibody-positive MS sera, antibody-negative healthy control sera, antibody-negative MS sera) were assayed, which were chosen based on results with the Antigenome Platform. Sera was considered antibody-positive in the ELISA assays if the OD was greater than two times the standard deviation above the mean of the healthy control samples. Outliers in the healthy control group were determined by Cauchy method using JMP software and

were excluded. One outlier was identified for the ELISAs KCTD17, PLCG1, and SPG20; no outliers were identified for the HGS ELISA. The following concentrations of purified serum IgG were used: 15 ug/mL for KCTD17, 5 ug/mL for PLCG1, 1 ug/mL for HGS, and 15 ug/mL of SPG20. Serum IgG was purified using 0.2 ml Nab Protein A/G Spin Columns (Thermo Fisher Scientific/Pierce Biotechnology, Rockford, IL) according to the manufacturer's protocol. The concentrations of purified IgG were determined using Pierce BCA Protein Assay Kits (Thermo Fisher Scientific/Pierce Biotechnology, Rockford, IL) according to the manufacturer's protocol.

References

1. Carroll WM. 2017 McDonald MS diagnostic criteria: Evidence-based revisions. *Mult Scler.* 2018;24(2):92-5.
2. Comi G, et al. Comparison of two dosing frequencies of subcutaneous interferon beta-1a in patients with a first clinical demyelinating event suggestive of multiple sclerosis (REFLEX): a phase 3 randomised controlled trial. Lancet Neurol. 2012;11(1):33-41.
3. De Stefano N, et al. Efficacy of subcutaneous interferon beta-1a on MRI outcomes in a randomised controlled trial of patients with clinically isolated syndromes. J Neurol Neurosurg Psychiatry. 2014;85(6):647-53.
4. Freedman MS, et al. Patient subgroup analyses of the treatment effect of subcutaneous interferon beta-1a on development of multiple sclerosis in the randomized controlled REFLEX study. J Neurol. 2014;261(3):490-9.
5. Polman CH, et al. Diagnostic criteria for multiple sclerosis: 2005 revisions to the "McDonald Criteria". *Ann Neurol.* 2005;58(6):840-6
6. Polman CH, et al. Diagnostic criteria for multiple sclerosis: 2010 revisions to the McDonald criteria. *Ann Neurol.* 2011;69(2):292-302.
7. R-Core-Team. R Foundation for Statistical Computing, Vienna, Austria. 2021.
8. Martin M. Cutadapt removes adapter sequences from high-throughput sequencing reads. EMBnet J. 2011(17):10-2.
9. Dobin A, et al. STAR: ultrafast universal RNA-seq aligner. *Bioinformatics.* 2013;29(1):15-21.
10. Evsyukova I, Somarelli JA, Gregory SG, Garcia-Blanco MA. Alternative splicing in multiple sclerosis and other autoimmune diseases. RNA Biol. 2010 Jul-Aug;7(4):462-73.
11. Anders S ea. HTSeq--a Python framework to work with high-throughput sequencing data. Bioinformatics. 2015;31(2):166-9.
12. Arslan T, et al. SubCellBarCode: integrated workflow for robust spatial proteomics by mass spectrometry. *Nat Protoc.* 2022;17(8):1832-67.
13. Uhlen M, et al. Proteomics. Tissue-based map of the human proteome. *Science.* 2015;347(6220):1260419.
14. Pomaznoy M, et al. GOnet: a tool for interactive Gene Ontology analysis. *BMC Bioinformatics.* 2018;19(1):470.
15. Vita R, et al. The Immune Epitope Database (IEDB): 2018 update. *Nucleic Acids Res.* 2018.
16. Subramanian A, et al. Gene set enrichment analysis: A knowledge-based approach for interpreting genome-wide expression profiles. *PNAS.* 2005.

Supplementary Table 1. Quality of phagemid cDNA library. Results of sanger sequencing analysis for phagemid inserts derived from the four libraries, PBMC, Astrocytes, HEP-2 cells, and white brain matter (brain). The number of phagemid inserts analyzed (Phage inserts surveyed), the percent of inserts that are open reading frames (ORF), the percent of inserts that encode a protein as opposed to a non-coding RNA (Protein), and the size of the insert (Size) are shown.

Supplementary Figures and Tables

Library	Phage Inserts Surveyed	ORF (%)	In-frame (%)	Protein (%)	Size (bp ±SD)
PBMC	19	84	84	73	189 ± 48
Astrocytes	38	82	71	42	221 ± 59
HEp-2	79	84	82	41	182 ± 57
Brain	130	72	68	27	256 ± 59

Supplementary Table 1. Quality of phagemid cDNA library. Results of sanger sequencing analysis for phagemid inserts derived from the four libraries, PBMC, Astrocytes, HEP-2 cells, and white brain matter (brain). The number of phagemid inserts analyzed (Phage inserts surveyed), the percent of inserts that are open reading frames (ORF), the percent of inserts that encode a protein as opposed to a non-coding RNA (Protein), and the size of the insert (Size) are shown.

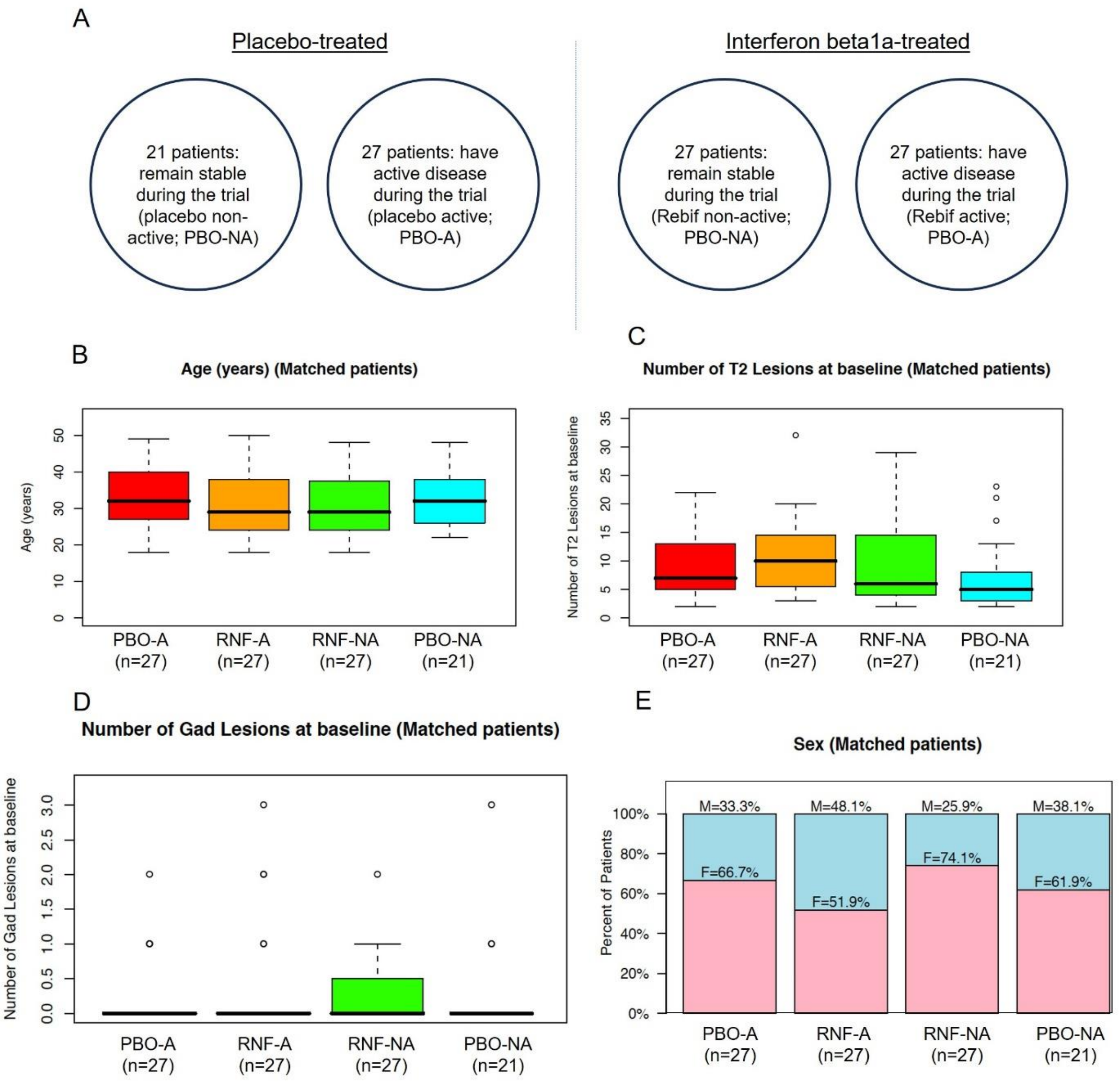

Supplementary Figure 1. Multiple sclerosis sample selection. (A) Illustration of the planned sample sizes and description of the 4 patient subgroups from REFLEX. (B-D) The boxplots show the distributions of (B) age (C) T2 Lesions, and (D) gadolinium (Gad)-positive lesions across subgroups and final sample sizes after selecting patients using propensity score matching. On the boxplots, the dark lines indicate median values; the boxes represent the middle 50% of data with the bottom and top of the box representing the first and third quartiles (interquartile range, IQR); the whisker lines above and below the boxes represented the largest and smallest values that are not considered to be outliers; outliers are values that are 1.5 times the IQR below the first quartile or above the third quartile and are denoted with an open circle. (E) The proportion of males and females in each subgroup.

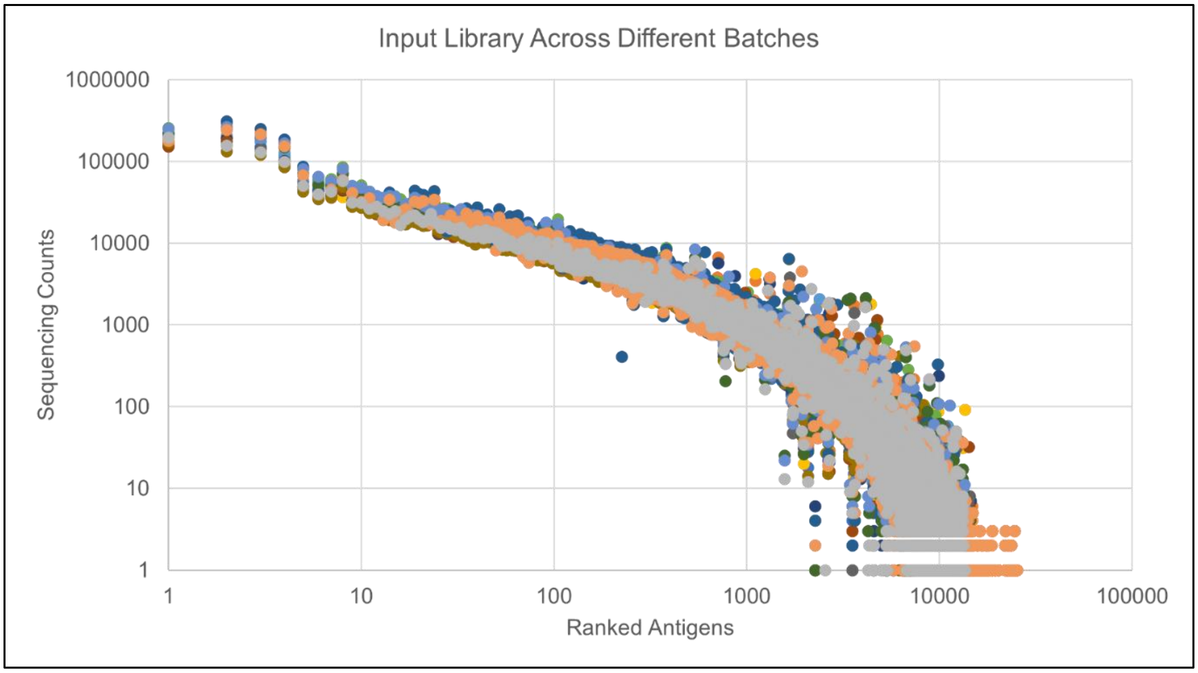

Supplementary Figure 2. Scatter plot showing sequencing counts for human proteins contained in the Input Library across a representative ten batches. Each dot represents a protein. The y-axis shows sequencing counts, and the x-axis shows the number of human proteins mapped in the libraries, ranked based on the Input Library sample from one batch. Color of dots indicate to which batch the dots correspond.

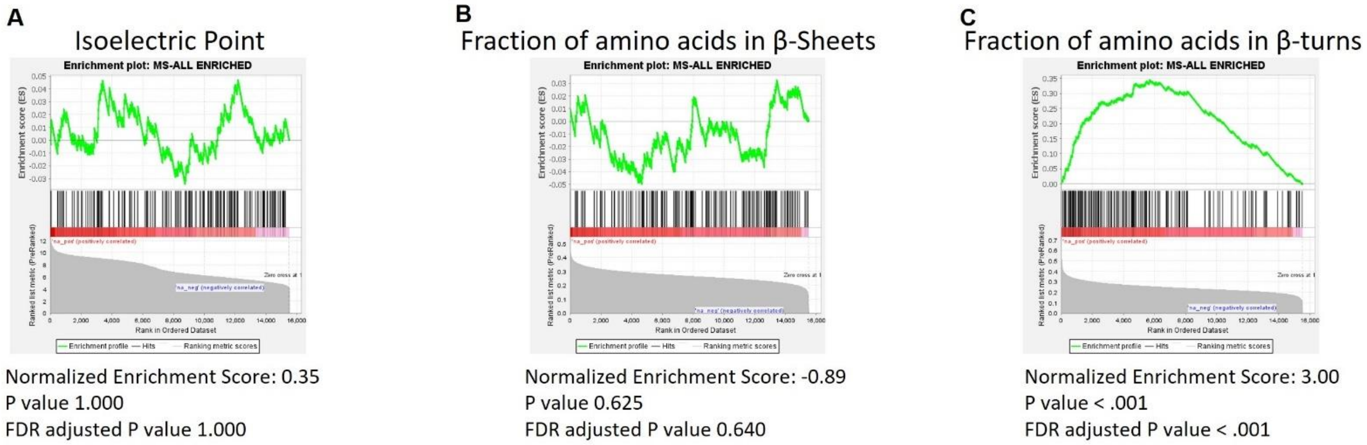

Supplementary Figure 3. (A-C) Protein structure properties as labeled. Values are sorted in descending order; the grey curve indicates the values for all proteins included, and the black vertical lines indicate the placement of the 166 CIS-enriched autoantigens in the ranked list. The green curve indicates the enrichment score. The red and blue color gradient represents positive (red) to negative (blue) values.

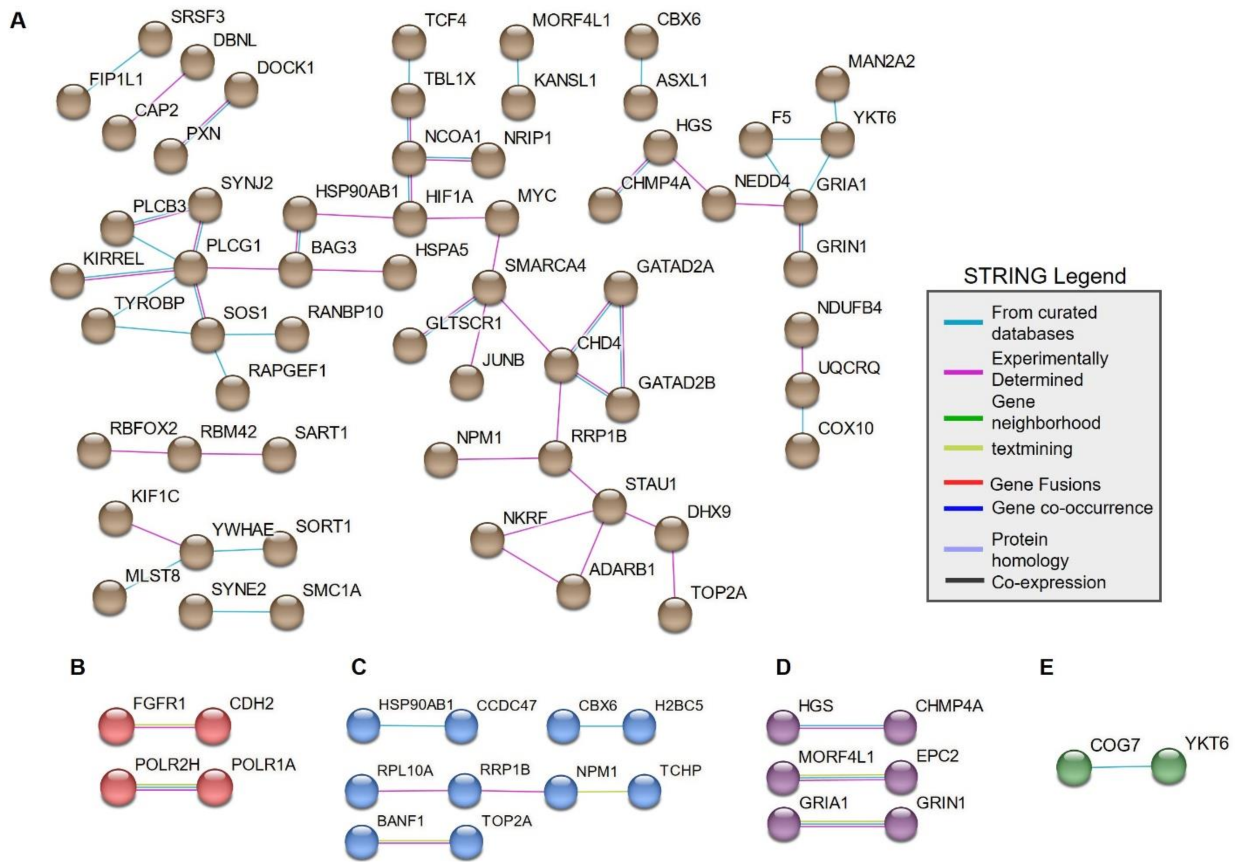

Supplementary Figure 4. Physical protein-protein interaction subnetwork construction. A protein-protein interaction subnetwork of autoantibody targets involved in physical complexes is shown for **(A)** The 166 CIS-enriched autoantigens selected by at least 10% of CIS patients, **(B)** PBO-A-enriched autoantigens selected by at least 10% of CIS patients, **(C)** PBO-NA-enriched autoantigens selected by at least 10% of CIS patients, **(D)** RNF-A-enriched autoantigens selected by at least 10% of CIS patients, **(E)** RNF-NA -enriched autoantigens selected by at least 10% of CIS patients. Where circles represent antigens, colored according to subgroup, and the connecting lines represent physical interactions between antigens, colored according to the type of data the information is derived from. Legend indicates what the color of the connecting lines represents. Autoantigens not involved in physical interactions are omitted.
